# FoldX force field revisited, an improved version

**DOI:** 10.1093/bioinformatics/btaf064

**Published:** 2025-02-06

**Authors:** Javier Delgado, Raul Reche, Damiano Cianferoni, Gabriele Orlando, Rob van der Kant, Frederic Rousseau, Joost Schymkowitz, Luis Serrano

**Affiliations:** Centre for Genomic Regulation (CRG), The Barcelona Institute for Science and Technology, Dr. Aiguader 88, Barcelona 08003, Spain; Centre for Genomic Regulation (CRG), The Barcelona Institute for Science and Technology, Dr. Aiguader 88, Barcelona 08003, Spain; Centre for Genomic Regulation (CRG), The Barcelona Institute for Science and Technology, Dr. Aiguader 88, Barcelona 08003, Spain; Switch Laboratory, VIB Center for Brain and Disease Research, VIB, 3000 Leuven, Belgium; Switch Laboratory, Department of Cellular and Molecular Medicine, KU Leuven, 3000 Leuven, Belgium; Switch Laboratory, VIB Center for AI & Computational Biology, VIB, 3000 Leuven, Belgium; Switch Laboratory, VIB Center for Brain and Disease Research, VIB, 3000 Leuven, Belgium; Switch Laboratory, Department of Cellular and Molecular Medicine, KU Leuven, 3000 Leuven, Belgium; Switch Laboratory, VIB Center for AI & Computational Biology, VIB, 3000 Leuven, Belgium; Switch Laboratory, VIB Center for Brain and Disease Research, VIB, 3000 Leuven, Belgium; Switch Laboratory, Department of Cellular and Molecular Medicine, KU Leuven, 3000 Leuven, Belgium; Switch Laboratory, VIB Center for AI & Computational Biology, VIB, 3000 Leuven, Belgium; Switch Laboratory, VIB Center for Brain and Disease Research, VIB, 3000 Leuven, Belgium; Switch Laboratory, Department of Cellular and Molecular Medicine, KU Leuven, 3000 Leuven, Belgium; Switch Laboratory, VIB Center for AI & Computational Biology, VIB, 3000 Leuven, Belgium; Centre for Genomic Regulation (CRG), The Barcelona Institute for Science and Technology, Dr. Aiguader 88, Barcelona 08003, Spain; Universitat Pompeu Fabra (UPF), Barcelona 08002, Spain; ICREA, Pg. Lluis Companys 23, Barcelona 08010, Spain

## Abstract

**Motivation:**

The FoldX force field was originally validated with a database of 1000 mutants at a time when there were few high-resolution structures. Here, we have manually curated a database of 5556 mutants affecting protein stability, resulting in 2484 highly confident mutations denominated FoldX stability dataset (FSD), represented in non-redundant X-ray structures with <2.5 Å resolution, not involving duplicates, metals, or prosthetic groups. Using this database, we have created a new version of the FoldX force field by introducing pi stacking, pH dependency for all charged residues, improving aromatic–aromatic interactions, modifying the Ncap contribution and α-helix dipole, recalibrating the side-chain entropy of methionine, adjusting the H-bond parameters, and modifying the solvation contribution of tryptophan and others.

**Results:**

These changes have led to significant improvements for the prediction of specific mutants involving the above residues/interactions and a statistically significant increase of FoldX predictions, as well as for the majority of the 20 aa. Removing all training sets data from FSD [Validation FoldX Stability Dataset (VFSD) dataset] resulted in improved predictions from *R* = 0.693 (RMSE = 1.277 kcal/mol) to *R* = 0.706 (RMSE = 1.252 kcal/mol) when compared with the previously released version. FoldX achieves 95% accuracy considering an error of ±0.85 kcal/mol in prediction and an area under the curve = 0.78 for the VFSD, predicting the sign of the energy change upon mutation.

**Availability and implementation:**

FoldX versions 4.1 and 5.1 are freely available for academics at https://foldxsuite.crg.eu/.

## 1 Introduction

Although the field of protein structure has experienced a revolution with the appearance of sequence-based structure predictive methods like Alphafold (Park *et al.* 2022), it has shown a very weak or no correlation between its metrics and changes in protein stability or fluorescence on single mutations (Pak *et al.* 2023). In this context, there is still room for methods that can deal with such limitations by using experimentally determined protein structures, especially after the improvements that techniques such as CryoEM are achieving (Nakane *et al.* 2020), and calculating mutation effects by local side-chain transformations.

The FoldX software (Delgado *et al.* 2019) has been established as one of the principal standards for prediction of ΔΔG changes in stability and binding upon mutation. FoldX is a software based on a rigid-solid approach where the backbone doesn't change upon mutation. FoldX forcefield was trained with a dataset of changes in stability for 1000 experimentally measured point mutations (Guerois *et al.* 2002) and their corresponding crystal structures. Later on it was improved by introducing amino acid side-chain movement, along with water and metal prediction (Schymkowitz *et al.* 2005, Delgado *et al.* 2019), mutating DNA and RNA (Delgado *et al.* 2019), and providing a graphic interface (Van Durme *et al.* 2011).

FoldX has been used by many groups and has been independently compared to different methods, emerging as one of the most accurate force fields to predict changes in stability and/or binding upon mutation in many studies (Potapov *et al.* 2009, Kamisetty *et al.* 2011, Bednar *et al.* 2015, Brender and Zhang 2015, Pires and Ascher 2016, Broom *et al.* 2017, Buß *et al.* 2018, Usmanova *et al.* 2018, Grilo and Mantalaris 2019, Montanucci *et al.* 2019, Sapozhnikov *et al.* 2023, Zhou *et al.* 2023). However, since its conception as a force field, it has not undergone a major revision despite the fact that the number of 3D structures has increased dramatically since its conception, as well as the datasets for changes upon mutation for stability as ProTherm (Nikam *et al.* 2021) or VariBench, and for binding as Skempy (Jankauskaitė *et al.* 2019).

## 2 Materials and methods

See Supplementary data containing Supplementary Methods.

## 3 Results

Here, we leverage a 5556 mutant dataset of protein stabilities as described in the paper of Montanucci *et al.* (2019) and Nikam *et al.* (2021) to improve FoldX force field. We manually curated the database by removing X-ray structures >2.5 Å resolution, mutations that involve interaction with metals and/or prosthetic groups, duplicate ones, and those that cause large Van der Waals clashes (>6 kcal/mol). This resulted in a database with 2484 unique mutants, the FoldX stability dataset (FSD; Supplementary Table S1). Using this dataset, we checked for mutations affecting specifically the FoldX force field parameters, like the double salt bridge between carboxylates and guanidinium moieties, the helix dipole and Ncap contributions, aromatic–aromatic interactions, aromatic–pi stacking, side-chain entropy, and solvation parameters. Then we created subsets of these mutations where we explored variations of the FoldX corresponding parameters, and once identified an optimal value, we ran blindly the modified force field on a validation dataset (VFSD). We have also introduced new features like aromatic pi (McGaughey *et al.* 1998, Liao *et al.* 2013, Bootsma *et al.* 2019) that involves His, Arg, and aromatic side chains and expanded the pH dependence of stability to include other charged residues different from His. Finally, we have recalibrated the geometry of hydrogen bonding by using a set of structures with <1.5 Å resolution.

The overall Pearson correlation for the FoldX version (v1) publicly available (https://foldxsuite.crg.eu/) for the VFSD after repairing the structures (see Supplementary Methods) is *R* = 0.693 and RMSE = 1.277, with a slope of 0.646 and an intersection with the *Y*-axis of 0.357 kcal/mol (Table 1). The corresponding modifications have been introduced stepwise, resulting in a correlation increase of FoldX predictions with experimental ΔΔGs, from *R* = 0.693 to *R* = 0.711, an increase of the correlation slope from 0.646 to 0.665, and RMSE from 1.277 to 1.252 (Table 1).

**Table 1.** FoldX modifications with their corresponding version name (v).

v	*R*	*S*	*I*	RMSE	Paragraph
v1	0.693	0.646	0.357	1.277	1
v2	0.693	0.644	0.355	1.281	3.3
v3	0.694	0.647	0.36	1.276	3.4
v4	0.696	0.647	0.352	1.273	3.5
v(2–4)	0.693	0.646	0.377	1.278	3.6
v5	0.695	0.644	0.357	1.278	3.7
v6	0.702	0.666	0.336	1.246	3.8
v7	0.703	0.665	0.338	1.247	3.9
v8	0.698	0.656	0.346	1.26	3.10
v9	0.702	0.662	0.334	1.251	3.11
v10	0.705	0.66	0.324	1.25	3.12
v10R	0.707	0.658	0.293	1.252	3.12
v10A	0.707	0.659	0.286	1.251	3.12
v10M	0.711	0.665	0.305	1.238	3.12

*R* (Pearson correlation) score, slope (*S*), interception *Y* coordinate of the linear fitting equation (1), and RMSE, ‘Paragraph’ references the section where the version is described. The results correspond to the 2275 mutants included in the VFSD.

Also, a general improvement is achieved for the prediction for most amino acid mutations with no specific bias for any aa (Supplementary Table S2). FoldX is freely available at https://foldxsuite.crg.eu/.

### 3.1 Curated mutant dataset (FSD)

To benchmark FoldX modifications and test each version, we used an extensive dataset compiled by Montanucci *et al.* (2022) that combines stabilities of different databases such as VariBench (Corretge and Nigretto 1984, Sasidharan Nair and Vihinen 2013, Broom *et al.* 2017, Banerjee and Mitra 2020), ProTherm (Nikam *et al.* 2021), and S2648 (Dehouck *et al.* 2011) resulting in 5556 non-curated mutations. The dataset was manually curated to remove factors that are not directly related to the force field calibration in order to have a benchmark set to evaluate the improvements made in the FoldX force field.

**Figure 1.**
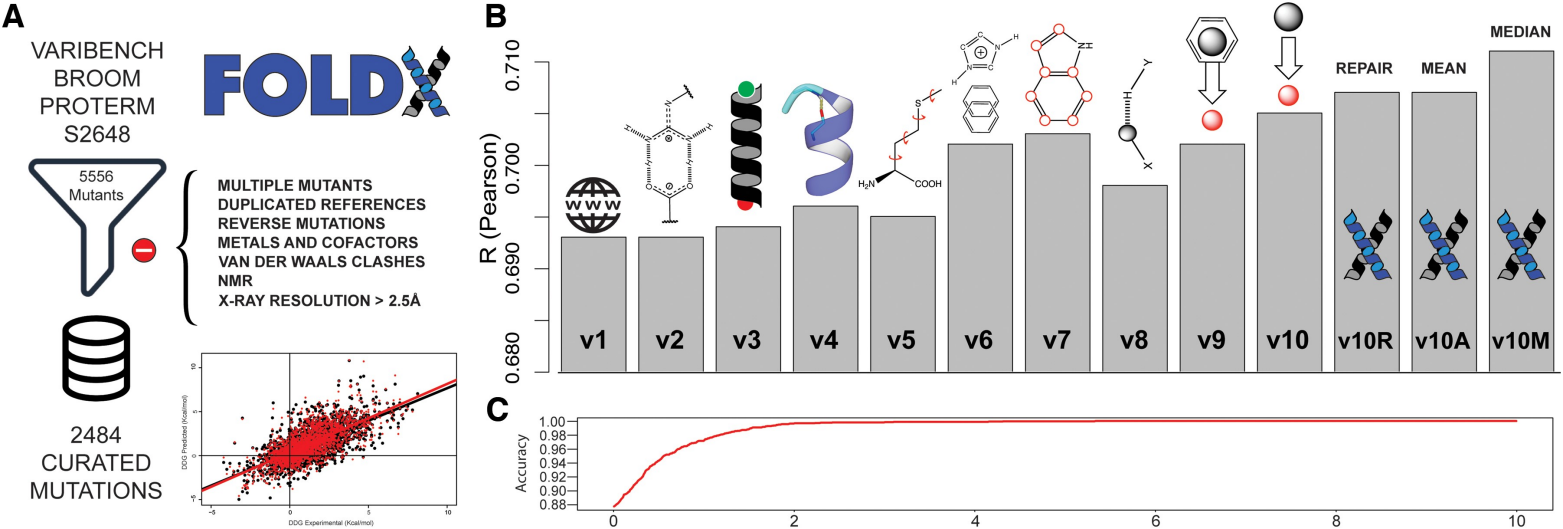
Summary of the FoldX optimization procedure. (A) Dataset curation requirements and global correlation for FoldX v1 versus v10. (B) *R* Pearson correlation for the different FoldX versions (v1–v10M) developed in this work (see Table 1 for version-specific correlation values). (C) Accuracy for v10, *X*-axis is FoldX error calculated in kcal/mol.

The dataset was first reduced by removing multiple mutations (they are likely to cause significant backbone conformational changes), duplicated reference WT structures, mutant duplicates, reverse mutations where the structure of the mutant restores the wild-type sequence (they involve, in many cases, strained structures that accommodate bulky residues with backbone displacements), mutations involving residues interacting with metals or cofactors, those that cause Van der Waals clashes >5 kcal/mol, NMR-determined structures, and those crystal structures having resolution above 2.5 Å (Fig. 1). Low-resolution structures should not be used to calibrate a force field since, by definition, they will have, in some cases, incorrect distances, dihedrals, etc. For example, if one plots the predicted FoldX energy for different SH3 structures, it is obvious that the worse the resolution, the worse the energy (Supplementary Fig. S1). And this is the reason why FoldX predictions, when including several structures of the same molecule, are better (Radusky and Serrano 2022). Similarly, a mutation that will introduce a large Van der Waals clash in a hydrophobic core will either unfold the protein or result in large backbone conformational changes. Since FoldX so far does not incorporate backbone moves, those mutants cannot be predicted. When there were duplicates analyzed at different temperatures, we selected those closer to 25°C. When there were two different experimental energies for the same mutant, the mean was calculated (Supplementary Table S1). In some cases, we looked for the original publications of the corresponding mutation and we corrected the ΔΔG values (Supplementary Table S3). These steps resulted in a manually curated dataset containing 2484 mutations (Supplementary Table S1), named FSD. We removed all calibration data from FSD, resulting in a Validation FSD (VFSD, 2275 mutations, Supplementary Table S1).

### 3.2 Modification of FoldX parameters

Calibration of the described FoldX features was done by selecting representative cases from the FSD of the property to modify (CALIBRATION subsets). Then we explore different values around the original FoldX value of the interaction to be modified. Then we run the optimum parameter on a VALIDATION subset (set of mutants affecting the interaction to be modified not used for CALIBRATION) and finally on the VFSD. In some cases when the changes affected many different amino acids, we first explored different parameters with a random subset, and then we validated the selected value with the rest of the VFSD.

Upon mutation, FoldX moves all neighboring residues. Although there are not many mutants directly affected by a parameter being changed (e.g. a double salt bridge), there are significantly more mutations located near a residue involved directly in that interaction. Therefore, we could expect changes in other mutations, which will tell us indirectly about the goodness of the change. The same principles of neighboring effects are equally valid for most force field adjustments requiring each time to look at the effect of a relatively small number of cases on all FSD mutants to see if there is an improvement or not. Obviously, changes in a specific interaction involving a small number of mutations will never increase the overall correlation parameters for the complete FSD by a large number.

For the CALIBRATION subset, we used as selection criteria an improvement in correlation (*R*) an improvement in intersection with the *Y*-axis (*I*) and/or slope of the full subset linear fitting (with priority given to an improvement in correlation factor *R*, or in specific cases to intersection with the *Y*-axis) defined as getting close to that of the same parameters found for the FSD. The reason being that although slope, or intersection, could be normalized for the whole FSD, we expect the correlation values for the corresponding mutants to become closer to those of the whole FSD; otherwise, a global normalization will result in incorrect predictions for specific mutations. After calibration, we look at the changes in the correlation parameters and determine RMSE values for the VALIDATION subsets and for the VFSD to measure the impact of the modification.

When no significant worsening of the RMSE for the VFSD is found, we accept the modification, or when the parameters are modified due to the use of more accurate data (e.g., hydrogen bond geometries).

For every new accepted run, the code was named with a different version name (Supplementary Table S1). A letter was added to v10 indicating if we run the same version on structures repaired with it (*R*), or run FoldX with the five runs option calculating the median (*M*) or average (*A*).

### 3.3 Double salt bridge (v2)

**Figure 2.**
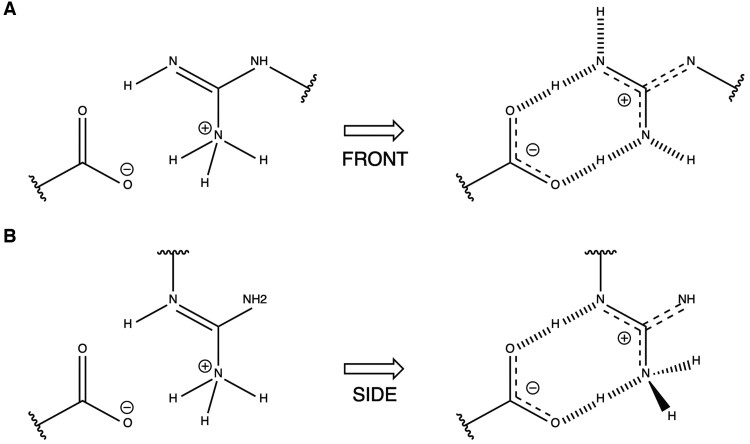
Delocalized double salt bridge between arginine side chain and a carboxylate group (aspartic or glutamic side chain or terminal oxygen). The negative charge is delocalized among two nitrogen groups and a carbon atom while the negative charge is delocalized between two oxygen atoms and a carbon atom. (A) FRONT case: the hydrogen bonds and hydrogen covalent bonds are also allowed to switch from nitrogen to oxygen propagating the delocalization of the electron cloud in a highly delocalized behavior. (B) SIDE case: the carbon CE cannot transpose as the hydrogen in FRONT case. This special bond architecture has a bigger energetic contribution than it was defined in v1 for both FRONT and SIDE.

Charged hydrogen bonds are established between the two oxygen atoms of an aspartic or glutamic and two-side-chain nitrogen of Arg (Fig. 2), or with the two-side-chain nitrogen of adenosine in DNA and RNA, and are a special class of hydrogen bonds. The reason is the delocalization of the electron cloud between both side chains.

There are 18 mutants involving FRONT and SIDE double salt bridges in the FSD (Supplementary Table S4). Comparison with experimental data shows that in the previous FoldX versions, the double salt bridge contribution was underestimated (Supplementary Fig. S2). From these 18 mutants, we extracted a CALIBRATION subset with five mutants and a VALIDATION subset with the remaining 13 (Supplementary Table S4). We then explored increasing contributions for double salt bridge bonds, multiplying the original value by 1, 1.25, 1.5, 1.75, 2.0. We found that the optimal factor was 1.5 which improves the CALIBRATION subset slope from 0.4 to 0.804 and correlation from *R* = 0.682 to *R* = 0.972 (Supplementary Fig. S2). With this correction, the RMSE for the VALIDATION subset goes from 1.521 (v1) to 1.467 (v2).

Modification of this contribution resulted in RMSE = 1.281, a Pearson *R* = 0.693, and a slope of 0.646 for the VFSD (Table 1). The Wilcoxon signed-rank test of v1 and v2 for the VFSD is statistically significant (*P* = 6.28 × 10^−3^).

### 3.4 Helix dipole (v3)

FoldX considers, as in AGADIR (Muñoz and Serrano 1994, 1995a, 1995b, Lacroix *et al.* 1998, Serrano 1998), that the alignment of the carbonyl and amino groups in a α-helix results in a partial positive charge at the helix N terminus and a partial positive charge at the helix C terminus. To model this, FoldX places a dummy atom with a positive charge of 0.5 at an equidistant distance of the first four nitrogen atoms of the amino groups and a negative charge of 0.5 at equidistant distance of the last four carbonyl oxygen groups of the helix. Then FoldX applies a modified Coulomb law (Eq. (1) in Supplementary Methods) to determine the electrostatic interaction between charges and the helix dipole by considering the distances between charges and the dummy atoms (Ncap and Ccap for the dummy atoms at the N- and C-termini of the helix, respectively).

In the publicly available v1, the equation used to determine the α-helix Ncap and Ccap dipole contributions was not dividing the electrostatic contribution by the square of the distance but simply by the distance. Therefore, an Aspartic at the Ncap of the helix had a disproportionate contribution compared with other good Ncap residues like Ser, Thr, and Asn. This can be seen by examination of a CALIBRATION subset (Supplementary Table S5) where we see an interception with the *Y*-axis of −3.02 kcal/mol. Correction of the square of the distance (Eq. (1) in Supplementary Methods) improved significantly the interception with the *Y*-axis (*I* =−1.4 kcal/mol), reduced slightly the correlation from *R* = 0.799 (v1) to *R* = 0.788 (v3) (Supplementary Fig. S3) and improved the RMSE from 3.044 (v1) to RMSE = 1.423. The reason we do not get an intercept close to zero is that, in this version, we still underestimate the Ncap contribution of Asn, Ser, and Thr (see below and Supplementary Fig. S3). We then checked the effect on a VALIDATION subset involving 56 mutations, with an absolute energy change for the FoldX term helix dipole >0.3 kcal/mol (Supplementary Table S5). By using the corrected equation, we improve the correlation from *R* = 0.640(v1) to *R* = 0.717 (v3) and from RMSE = 1.675 (v1) to RMSE = 1.211(v3).

The overall correlation for the VFSD is *R* = 0.694 and RMSE = 1.276. The Wilcoxon signed-rank test of v2 and v3 for the VFSD is statistically significant (*P* = 8.35 × 10^−4^).

### 3.5 Ncap (v4)

It is experimentally known that a Ser, Thr, Asn, or Asp at the Ncap (Richardson and Richardson 1988) of an α-helix whose side chain makes a hydrogen bond to the (Ncap + 3)N atom contributes significantly to the stability of the helix (Serrano and Fersht 1989, Abagyan and Totrov 1994). FoldX already incorporated this by giving extra energy to that H-bond, but the analysis of the new extended database showed that it can be improved as observed using a mutant CALIBRATION subset (Supplementary Table S6 and Fig. S4). The CALIBRATION subset includes mutations of Thr to all favorable Ncap residues (Gly, Asn, Ser), except Asp (we calibrated the dipole contribution above), and to Ala as a reference. We increased the contribution of Ser, Asn, Thr to the Ncap by multiplying the H-bond energy contribution (1.32 kcal/mol) by 1.5 or 2. We sweep the following combinations: 1.5 for Ser, Asn, and Thr; 2 for Ser, Asn, and Thr; 1.5 for Ser, Asn, and 2 for Thr; 2 for Ser, Asn, and 1.5 for Thr. The combination of multiplying the H-bond energy contribution by 1.5 for Ser, Asn, and Thr by 2 resulted in the best parameters (FoldXv4). FoldXv4 improves with respect to v1 the slope from 0.529 to 0.961, the interception with the *Y*-axis from I= -0.766 to *I* = 0.151 and the Pearson correlation from *R* = 0.560 to *R* = 0.715 for the CALIBRATION subset (Supplementary Fig. S4). We then defined a VALIDATION subset containing 24 Ser, Thr, and Asn mutations to any amino acid excluding the CALIBRATION subset (Supplementary Table S6). FoldXv4 improves the Pearson correlation for this subset from *R* = 0.590 (v1) *R* = 0.750 (v4) and from RMSE = 1.850 (v1) to RMSE = 1.512 (Supplementary Fig. S4).

The overall correlation for the VFSD is *R* = 0.696 and RMSE = 1.273 kcal/mol (Table 1). The Wilcoxon signed-rank test of v3 and v4 for the VFSD is statistically significant (*P* = 2.08 × 10^−3^).

### 3.6 Integration of versions v2–v4 (v(2–4))

At this point, and since from now on all changes in FoldX imply introduction of new interactions, we integrated all the above changes incorporated until now (v2–v4), which predicted the complete VFSD with a slope of 0.646, *R* = 0.693, RMSE = 1.278 and *Y*-axis interception *I* = 0.377 (Table 1). The Wilcoxon signed-rank test of v4 and v(2–4) for the VFSD is statistically significant (*P* = 4.04 × 10^−10^).

### 3.7 Met side-chain entropy (v5)

FoldX has a clear tendency to favor Met introduction in the hydrophobic core of proteins when exploring stabilizing mutations, which does not correspond to the relative frequency of this residue versus Leu, Ile, Val, and Phe. This over-favorable contribution of Met can be seen (Supplementary Fig. S5) when looking at the Intercept of the CALIBRATION subset composed of mutations from Met to other hydrophobic or small nonpolar residues (Supplementary Table S7 and Fig. S5). FoldX’s prediction for this subset had an interception with the *Y*-axis of 1.17 kcal/mol (Supplementary Fig. S5), suggesting that in general we were overestimating the positive contribution of Met to stability. One possible reason could be that the entropic cost of fixing a Met residue (ΔS_Abagyan) obtained from Abagyan and co-workers original paper (Abagyan and Totrov 1994) was underestimated. In the classic FoldX version, the value of the parameter ΔS_Abagyan (Eq. (2) in Supplementary Methods) was 0.0051 for Met, and 0.0067333 for Gln. Gln has one less covalent side-chain bond with free rotation than Met and consequently should pay a smaller side-chain entropic cost upon fixing it than Met. Thus, we adjusted the entropic cost of fixing a Met side chain by testing the value ΔS_Abagyan of Gln, Glu (0.0055000), Arg (0.0071000), and Lys (0.0073667) instead of that of Met, expecting to reduce the intercept with the *Y*-axis. The best entropic value was found to be that of Lys, which decreased the *Y*-axis intersection coordinate from I = 1.17 kcal/mol to *I* = 0.586 kcal/mol (Supplementary Fig. S5). This value was tested in a VALIDATION subset containing 29 mutations, where Met was changed to any hydrophobic amino acid (Supplementary Table S7). It resulted in decreasing the intercept from *I* = 0.803 to *I* = 0.488 with RMSE going from 1.344 (v(2–4)) to RMSE = 1.320 (v5).

Running FoldXv5 over the VFSD resulted in *R* = 0.695 with RMSE = 1.278. The Wilcoxon signed-rank test of v(2–4) and v5 for the VFSD is statistically significant (*P* = 5.76 × 10^−11^).

### 3.8 Pi–pi interactions and PH dependency (v6)

#### 3.8.1 Pi–pi interactions

Theoretical and experimental analyses on pi–pi stacking for the interaction between aromatic rings, or between a His ring or the guanidine group of Arg with aromatic rings, have shown that they could contribute significantly to protein binding and stability (McGaughey *et al.* 1998, Brylinski 2018). FoldX originally incorporated an electrostatic term to mimic the parallel or perpendicular stacking of residues capable of establishing pi–pi interactions. This was done by introducing a negative dummy dipole charge in the center of the aromatic ring and smaller dummy dipole charges at the side-chain carbon atoms of the ring. For arginine, we placed only a dummy atom with a net positive charge at the center of the guanidine group, and histidine was not included as a pi–pi interacting group. This resulted in very low interaction energies and not a very good correlation (*R* = 0.590) when using a CALIBRATION subset (Supplementary Fig. S6 and Table S8).

To properly introduce pi–pi stacking, we introduced an electrostatic dipole term based on theoretical and experimental analysis on pi–pi stacking for the interaction between aromatic rings and a His ring (we introduced a dummy atom in the center of the His ring), or the guanidine group of Arg, with aromatic rings, or with another Arg guanidine group (McGaughey *et al.* 1998, Brylinski 2018). To calculate the free energy contribution of the pi–pi stacking, we first determined the distance between two dummy atoms placed at the center of the histidine, aromatic, and/or guanidinium groups using 5 Å as cutoff to not calculate the interaction between the side chains. Then we determined the angle between the planes of the aromatics, histidine, and/or guanidine rings. For the imidazole ring in the histidine and guanidine groups in Arg interacting with an aromatic ring, we only calculated an energy contribution if the angle between the planes of the two interacting rings/groups is close to flat (angles above 30° are not considered). We did a similar thing for the interaction between two guanidine groups. We then assign an optimum interaction energy of 2.5 kcal/mol (E_opt) except for the interactions involving a Phe, which are 0.25 weaker (in the case of two Phe, they are 0.5 kcal/mol weaker) (McGaughey *et al.* 1998, Brylinski 2018). We then corrected the energy by applying a factor (ϑ) for the angle between the two rings (Eq. (3) in Supplementary Methods), or between a ring and a guanidine group and an exponential correction factor based on the distance of the two dummy groups (Eq. (4) in Supplementary Methods). If the two residues involved are Phe, Tyr, and/or Trp, we used only the distance between the dummy atoms to correct the interaction energy (ϑ = 1).

Introduction of the new way of calculating the pi–pi interaction resulted in an improved correlation going from *R* = 0.590 (v5) to *R* = 0.736 (v6) for the CALIBRATION subset (Supplementary Table S8 and Fig. S6). A VALIDATION subset containing 327 Phe, His, Tyr, and Trp mutations to any amino acid (Supplementary Table S8) showed an improvement in the correlation going from *R* = 0.642 (v5) to *R* = 0.667 and RMSE = 1.532 (v5) to RMSE = 1.489. Representative examples of pi–pi interactions can be found in Supplementary Fig. S7.

#### 3.8.2 pH dependency

It is known that experimentally determined pKas are usually determined in water and that protonatable groups in proteins are highly influenced by a different chemical surrounding than in solution. For that reason, in v7 we calculate environmental electrostatically modified pKas considering the sum of all favorable and unfavorable electrostatic interactions for the protonatable moiety (Eq. (5) in Supplementary Methods).

Originally, FoldX used the pKas of His and Cys to determine their protonation status, assigning a fixed pKa value of 6.8 to His and of 8.5 to Cys, but did not do the same for Asp, Glu, Lys, Arg, and Tyr. To solve this, we included the ionization status of Asp, Glu, Lys, Arg, and Tyr according to experimentally measured pKas (Supplementary Table S9) (Pahari *et al.* 2019) that are environmentally corrected by the electrostatic interactions with the surrounding residues (Eq. (5) in Supplementary Methods). Then the electrostatic contribution of an atom to the free energy takes into account its degree of ionization at a selected pH (Eq. (6) in Supplementary Methods) and the energy cost for ionization related to the solvent accessibility (ς) of the corresponding atom (Eq. (7) in Supplementary Methods).

**Figure 3.**
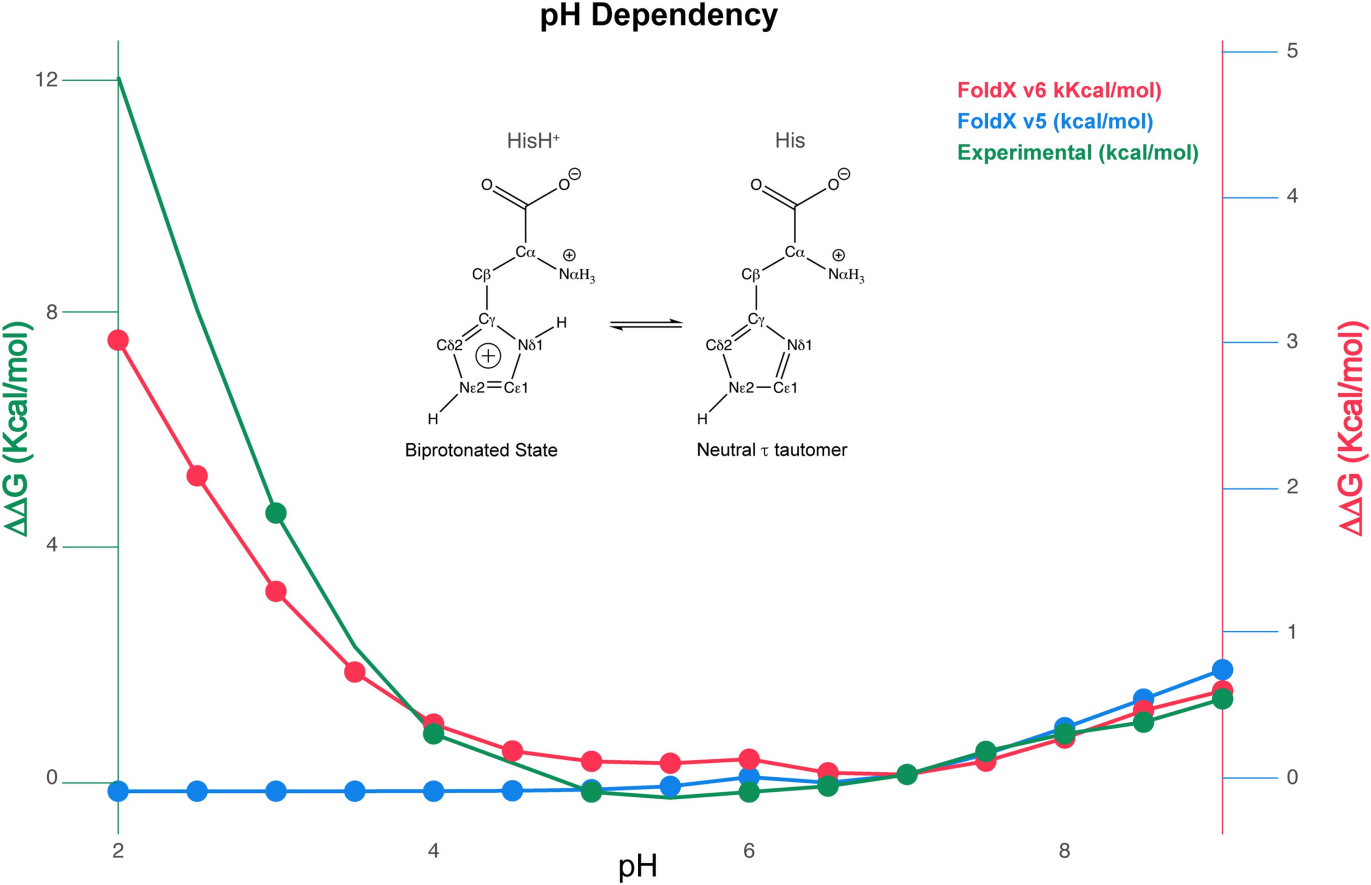
pH dependency of the ΔΔG changes of mutation of Barnase His18 to alanine. The new equation to calculate pH dependency reproduces the experimental pH dependence on stability for Barnase that in its wild-type form has a histidine in position 18. ΔΔGs were calculated by the following equation ΔΔG = ΔGpH-ΔGpH = 7. (Blue) FoldXv6 stability prediction lacking the new pH dependence modification (units in right *Y*-axis). (Red), FoldXv7 stability prediction with the new pH modification. (Green), Experimental ΔΔGs data (units in left *Y*-axis).

To test the prediction of protein stability upon pH, we used data from Barnase (Loewenthal *et al.* 1992, Pace *et al.* 1992). The energetic calculations fit much better for v6, the experimental trend of the energy curve, than for v5 (Fig. 3). FoldXv6 including the pi–pi stacking and the pH dependence for the VFSD subset resulted in *R* = 0.702 with RMSE = 1.246 (Table 1). The Wilcoxon signed-rank test of v5 and v6 for the VFSD is not statistically significant (*P* = .373).

### 3.9 Solvation parameters (v7)

In a comprehensive analysis for experimental and theoretical analysis of the desolvation cost of amino acids, Wolfenden (2007) described that Trp could have different behaviors depending on the experiment. Since FoldX was developed before this work, we decided to reexamine the solvation parameters by comparing the FoldX’s side-chain ΔG desolvation cost of the 20 aa with experimental and theoretical values determined from different experiments (Wolfenden 2007) (Supplementary Table S10). We found that the best correlation (*R* value and slope) is with the theoretical pentapeptide ΔG values for transfer from water to wet octanol (WW), obtained after adjustment of experimental values for the estimated effects of occlusion by neighboring residues (Wimley *et al.* 1996) (*R* = 0.921, see Supplementary Fig. S8), followed by the theoretical values obtained from the statistical tendency of amino acids to be in a transmembrane helix (Hessa *et al.* 2005) (*R* = 0.894, see Supplementary Fig. S8).

If we focus on the best correlation with the WW data (Supplementary Fig. S9), we see three residues (Asp, Glu, and Trp), deviating more from the ideal correlation. In the case of Trp, we are slightly underestimating its hydrophobicity, and the opposite happens for Asp and Glu. To solve this, we added −0.2 kcal/mol to all Trp side-chain atoms except for the polar side-chain NH group. For Asp, we reduced the hydrophobic desolvation of the CG to zero, and for Glu, the CD to zero and the CG by 0.30 kcal/mol, assuming that being so close to the charged groups, they will contribute little to hydrophobic desolvation. This positioned Trp, Asp, and Glu in line with the other aa; see Supplementary Fig. S9. Then we tested the correlation between predicted and experimental values for DX (Asp to any aa, 172 mutants; Supplementary Table S11), EX (Glu to any aa, 158 mutants; Supplementary Table S12), and WX (Trp to any aa, 38 mutants; Supplementary Table S13). In the case of Asp and Glu, we did not see an improvement in correlation parameters (Supplementary Fig. S10A and B), but the correction done for Trp improved the prediction for the WX subset (*R* = 0.580 (v6) to *R* = 0.594 (v7); Supplementary Fig. S10C). Thus, we only kept the changes in solvation of Trp for v8.

Testing v7 and v8 for VFSD resulted in *R* = 0.703 with RMSE = 1.247 (Table 1). The Wilcoxon signed-rank test of v6 and v7 for the VFSD is statistically significant (*P* = 8 × 10^−4^).

### 3.10 Hydrogen bonding (v8)

By looking at Xtal structures with a resolution <1.5 Å resolution, there is enough data to refine the H-bond geometric parameters and to distinguish between H-bonds involving backbone atoms or involving backbone and side-chain or side-chain side-chain atoms, the third group being more tolerant with the angle boundaries (Table 2). This was not possible for the first releases of FoldX (v1) due to the lack of experimental structural data at this resolution.

**Table 2.** H-bond angle experimentally measured ranges fenced by maximum, minimum, and optimal values.

	FoldXv1	FoldXv8		
	ALL-ALL	BB-BB	BB-SC	SC-SC
maxFreeProtDon	180	180	180	180
minFreeProtDon	115	115	90	90
optMaxFreeProtDon	160	170	170	170
optMinFreeProtDon	145	145	120	115
maxProtFreeAcc	155	135	180	180
minProtFreeAcc	80	75	70	70
optMaxProtFreeAcc	110	115	145	160
optMinProtFreeAcc	90	90	90	90
optDihed	130	145	160	170

FoldX v8 contains H-bond geometry boundaries for three groups of atoms: BB-BB, backbone-backbone; BB-SC, backbone-side-chain; SC-SC, side-chain-side-chain. maxFreeProtDon, maximum value of freeProtDon; minFreeProtDon, minimum value of freeProtDon; optMaxFreeProtDon, maximum optimum value of freeProtDon; optMinFreeProtDon, minimum optimum value of freeProtDon; maxProtFreeAcc, maximum value of protFreeAcc; minProtFreeAcc, maximum value of protFreeAcc; optMaxProtFreeAcc, maximum optimum value of protFreeAcc; optMinProtFreeAcc, minimum optimum value of protFreeAcc; optDihed, optimum value of dihed.

**Figure 4.**
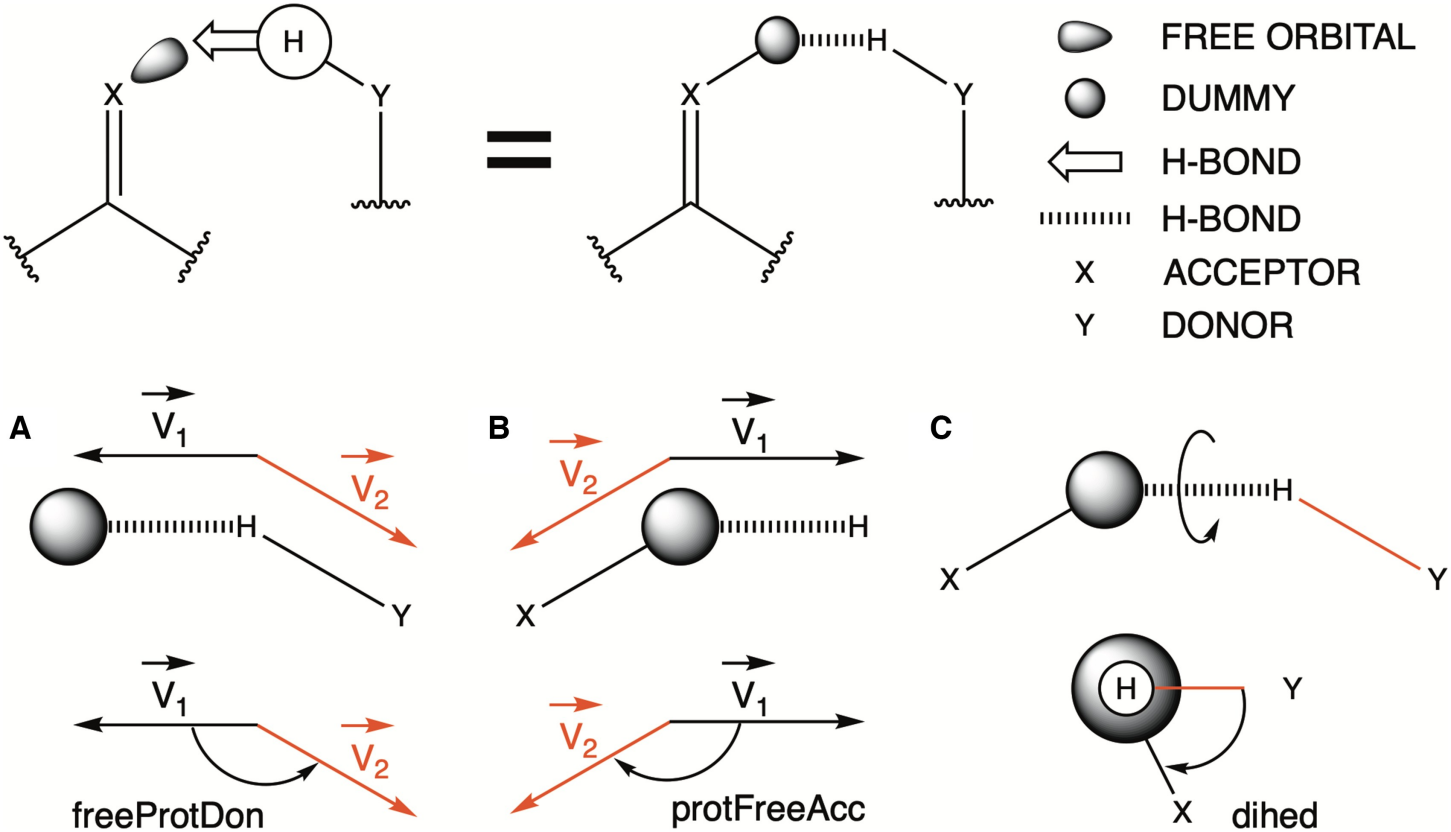
H bonding description and experimentally observed geometries. (A) freeProtDon is the angle between the vector of the proton of the donor and the free orbital of the acceptor represented as dummy atoms, and the vector between the proton and the donor atom. (B) protFreeAcc is the angle between the vector of the free orbital of the acceptor represented as dummy atoms and the proton of the donor, and the vector between the acceptor atom and its free orbital. (C) dihed is the dihedral angle between the donor atom, the proton, the free orbital dummy, and the acceptor atom.

FoldX weights the energy contribution of a neutral H-bond by the angles between the donor and acceptor, as well as between the neighbor atoms of donor and acceptor by calculating three angles: freeProtDon, protFreeAcc, and dihed (Fig. 4). If the calculated values are above or below the maximum-minimum ranges for the angle’s geometry, the atomic interactions will not be considered as H-bond and consequently its energy will not be computed. The more it deviates from the optimal values, the more energetically penalized the H-bond will be.

Changing the H-bond parameters (v8) reduced the overall correlation parameters for VFSD from *R* = 0.703 and RMSE = 1.247 (v7) to *R* = 0.698 and RMSE = 1.260 (v8) as could be expected since we have become more rigorous in accepting H-bonds and the structures used for the mutants have a resolution worse than 1.5 Å (Supplementary Fig. S11). The Wilcoxon signed-rank test of v7 and v8 for the VFSD is not statistically significant (*P* = .313).

### 3.11 Volume buried (v9)

#### 3.11.1 Aromatics volume buried

FoldX calculates the burial of an atom by determining the neighbor atoms, their volume and their distance. We found that aromatic rings, where the atoms of the side chain are closer than in other amino acids, increased the burial of a neighbor atom excessively with respect to the same atomic distance between non-ring atoms with similar volumes. Therefore, they will proportionally bury a larger surface. To correct for this, we modified the volume of the ring atoms selecting the best multiplying factor among: 0.8, 0.9, 1.0, 1.1, and 1.2. To find the best values, we took all the mutations Phe, Tyr, and Trp to any amino acid resulting in 261 mutants (Supplementary Table S14). We split the dataset into two parts: one on approximately half of the dataset taken at random (CALIBRATION subset, 130 mutants) and then blindly on the remaining mutants (VALIDATION subset, 131 mutants). We computed the mutations by comparing them with v8. We found that the best multiplying factor was 0.9 with the correlation going from *R* = 0.482 (v8) to *R* = 0.516 (v9) with RMSE = 1.904 (v8) to RMSE = 1.839 (v9) for the training subset (Supplementary Fig. S12A) and from *R* = 0.542 (v8) to *R* = 0.574 (v9) with RMSE = 1.776 (v8) to RMSE = 1.683 (v9) for the VALIDATION subset (Supplementary Fig. S12B).

The overall correlation for VFSD resulted in *R* = 0.702 with RMSE = 1.251 (Table 1). The Wilcoxon signed-rank test of v8 and v9 for the VFSD is statistically significant (*P* = 9.27 × 10^−7^).

#### 3.11.2 Minimum volume buried (v10)

FoldX uses a minimum buried volume, which is that of a fully solvent-exposed amino acid, and a maximum buried volume, which is that of a fully buried amino acid. Then it calculates the percentage of accessibility by comparing the calculated value with that of the minimum and maximum buried volumes (Eqs (8) and (9) in Supplementary Methods). Now we found on many occasions that a fully solvent-exposed hydrophobic side chain could contribute to the total energy just because of the conformation of the backbone that results in a calculated buried volume above the minimum. Also looking at specific aa we found that the Pro ring atoms had a minimum buried volume too low compared with other amino acids. Minimum proline volumes buried are 157 for CB, 147 for CG, and 170 for CD, while for Phe, His, and Tyr are 197 for CB and 209 for CG. These values are not realistic since CG and CD atoms are part of a ring, and therefore, they will bury each other at least like in ring containing residues.

To solve these issues, we increase the minimum buried volume of all atoms by adding the following values: 5, 10, and 15. And in the case of Pro, we increased the Pro minimum buried volume for the CB to that of Phe, Tyr and His, and the same for the CD since it is equivalent to the CB in terms of proximity to the backbone. Regarding the CD, we increased it to keep the 10 original differences with the CB. To test the effect of changing the Pro and overall minimum volume buried, we first made a subset including mutations to Pro or from Pro [87 mutations (Supplementary Table S15)]. We found that the changes to Pro improve prediction for the subset with Pearson correlation going from *R* = 0.624 (v9) to *R* = 0.651 and from RMSE = 1.692 (v9) to RMSE = 1.592 (Supplementary Fig. S13), independently of the increase in the minimum buried volume of all atoms (5, 10, and 15 Å^2^). To decide which correction to the minimum volume we should apply, we repaired WT PDB structures as we did with the original v1. We then did a normal FoldX run (v10R), and since running FoldX five times improves the predictions (Radusky and Serrano 2022), we also run it, setting the FoldX parameter numberOfRuns to 5, and then calculate the mean (v10A) and median (v10M) of the outputs from the five runs (see Supplementary Methods) over the whole FSD.

We found that the best correction for the minimum volume buried was by 10 (*R* = 0.704, RMSE = 1.287); even when 5 (*R* = 0.706, RMSE = 1.289) had similar *R* and RMSE values for v10R than 10 (*R* = 0.705, RMSE = 1.290), 10A and 10M got much better for both metrics (Fig. S14). We fixed the minimum occupancy to 10 for v10. The Wilcoxon signed-rank test of v9 and v10 for the VFSD is statistically significant (*P* = 8.66 × 10^−28^).

**Figure 5.**
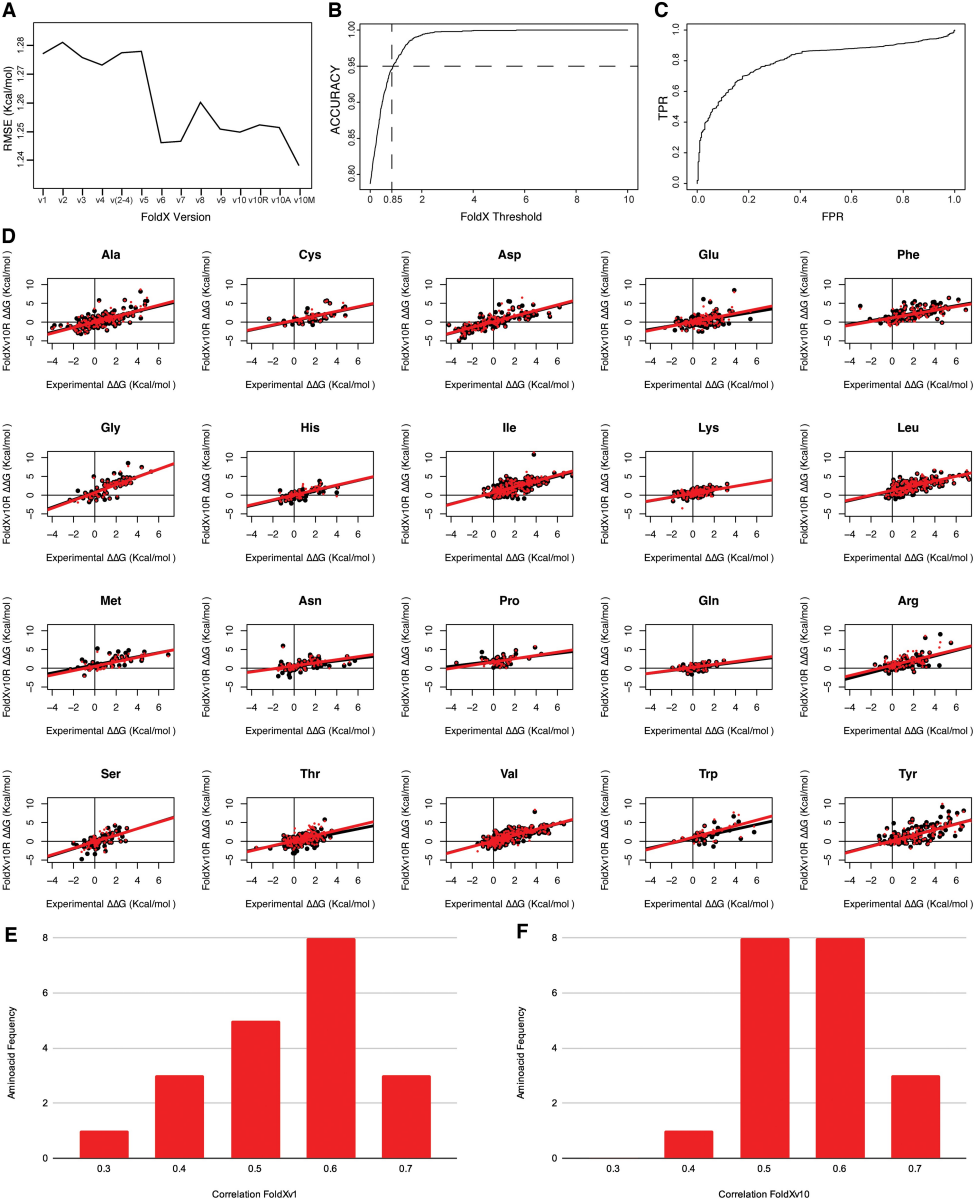
(A) RMSE values in kcal/mol for VFSD along the different version modifications. (B) FoldX ΔΔG predictions accuracy versus FoldX error for v11M, 0.95 accuracy is achieved for 0.85 kcal/mol FoldX’s energy threshold. (C) ROC curve for the VFSD (AUC = 0.78). (D) Correlation per residue comparison between experimental energy versus predicted energies for v1 (black), v11R (red). Correlations for each residue (Res) were done taking mutations Res to any amino acid from the FSD (Supplementary Table S3). (E) Histogram of *R* correlation values for experimental versus FoldXv1. (F) Histogram of *R* correlation values for experimental versus FoldXv10.

FoldX 10 version, using VFSD, results in *R* = 0.702 and RMSE = 1.252 kcal/mol. If we use the median of the five runs, we see an improvement in the correlation value, from *R* = 0.711 (v10M) and RMSE RMSE = 1.238 kcal/mol (Table 1). If we use the median of five runs, we get *R* = 0.709 (v10M)) and RMSE =1.277 kcal/mol (Fig. 5A). The Wilcoxon signed-rank test of v1 and v10R for the VFSD is statistically significant (*P* = 2.64 × 10^−20^).

We then looked at the prediction for the 20 aa to see if any of them was deviating from the general behavior (Supplementary Table S2). In general, we see an improvement in the slope and coefficient *R* for mutations for the 20aa (Fig. 5D; Supplementary Table S2), and we do not see any case where the predictions will be at the edge of the overall correlation (Table 1). Also, the *R* correlation values per amino acid improved from FoldXv1 (Fig. 5E) to FoldXV10 (Fig. 5F), shifting the histogram to values closer to 0 and reducing the frequency of amino acids with low *R* values and subsequently increasing the frequency of amino acids with high correlation values.

### 3.12 FoldX accuracy predictions

The RMSE numerical error value of the final version 10 for VFSD is 1.252 kcal/mol (it goes down to 1.238 when using the median of five runs, V10M). To assess FoldX accuracy, we varied what we denominated FoldX error from 0 to 10 (it means adding or subtracting a fixed value to the FoldX predicted energy). The uncertainty of FoldX ΔΔGs predictions (v10M) is evaluated by defining a correct prediction as one where the experimental ΔΔG falls within the predicted ΔΔG of FoldX ± FoldX error (Fig. 5B). From this analysis, we can extrapolate that 95% accuracy is achieved when what we denominate as FoldX error is 0.85 kcal/mol on the VFSD dataset. This number is very similar to the one previously published with a database of 1000 mutants (0.8 kcal/mol, Guerois *et al.* 2002). Besides the absolute value, what is important in protein design is the prediction of the sign of the ΔΔG of a mutation. To see how well FoldX does in this respect, we estimated an ROC curve over the v10M predictions over the VFSD dataset, revealing an area under the curve (AUC) of 0.78 (Fig. 5C).

## 4 Discussion

Proper calibration of a protein force field requires a clean experimental dataset free of exogenous elements that are not strictly related to the parameters to be estimated. Thus, mutations involving interactions with cofactors, metals, RNA, DNA, sugars, and modified amino acids should not be included unless we are calibrating the interactions with these atoms and/or molecules. Similarly, as much as possible, mutations that involve large backbone moves should not be included unless we are calibrating an algorithm that will do backbone moves. Thus, mutations that introduce large Van der Waals clashes or the use of mutant structures as a reference where a large conformational strain to alleviate clashes has been introduced should not be used. To improve the FoldX force field, we have chosen the database described in (Montanucci *et al.* 2019, Nikam *et al.* 2021) used for the ddGun predictor. We have thoroughly cleaned the database removing all mutations of the kind described above. After this cleaning, we obtained a dataset (FSD) with 2484 mutants.

The original FoldX software available on the web predicted those mutants with a slope of 0.738 and a correlation of *R* = 0.687, while the ddGun values for the same database had a slope of 0.44, an intercept of 0.199, and an *R* value of 0.574.

Having a clean, large dataset allowed us to improve some of the parameters present in the public version of FoldX, as well as introduce new features like the pi–pi stacking of Arg and His with aromatics and the pH dependence of the stability contributions of all protein amino acids that can be charged at a certain pH. For specific features, we selected a subset of representative mutations and tested how the modification of the parameter improves the prediction for the subset, thus avoiding overfitting, and afterwards with the 2275 mutants of the VFSD dataset. For general changes like the minimum volume buried of an atom, we tested different values on half the dataset and chose the best one, which was then tested on the other half.

As a result of the changes, we improved the overall prediction for the 2275 mutants, with the maximum correlation increase from *R* = 0.693 (RMSE = 1.277) for v1 to *R* = 0.711 (RMSE = 1.238) for v10M and the correlation slope rising from 0.646 to 0.665. The intersection with the *X*-axis is very close to zero, around *I* ∼ 0.3 kcal/mol. We also had an overall improvement for the majority of mutations involving individually each of the 20 amino acids.

Additionally, we calculated the delta-pLDDT scores for AlphaFold2 models for the FSD dataset by subtracting their pLDDT from that of the modeled wild-type sequence. We did the same calculation using FoldX ΔGs instead of pLDDTs over AlphaFold2 models (Supplementary Table S1). In both cases we observed no correlation with experimentally determined ΔΔGs (*R*=−0.02 for pLDDTs and *R* = 0.06 for FoldX ΔGs), corroborating the observation that AlphaFold2 models are not suitable for point mutation ΔΔG estimation (Pak *et al.* 2023).

The improvement in FoldX predictions shown here has been tested with high-quality X-ray structures, excluding mutations involving residues interacting with metals or cofactors. When running FoldX on protein structures with worse resolution, or quality, FoldX predictions worsen as shown by running it on the mutations from the Montanucci datatset (Montanucci *et al.* 2019, Nikam *et al.* 2021) done on NMR structures (Supplementary Table S16).

To avoid overfitting, we removed all training sets data from the FSD, resulting in a VFSD (2275 mutations). For this dataset, the correlation prediction improved from *R* = 0.693 (RMSE = 1.277) to *R* = 0.711 (RMSE = 1.238), when compared with FoldX old version.

If we look at the capability of FoldX in discriminating between stabilizing and destabilizing mutations, we get a 95% accuracy when we consider an error of 0.85 kcal/mol in the prediction energy of a mutant for FoldX. We find an AUC = 0.78 for the ROC curve for the agreement between the mutants’ experimental data sign and FoldX predictions, even for very small experimental values. Thus, the numerical error for FoldX is 1.277 kcal/mol, but the value of ∼0.8 kcal/mol previously described in the original FoldX paper (Guerois *et al.* 2002) can be safely used as an estimate of the error of FoldX calculations with 95% confidence.

## 5 Conclusions

Here, we present a new version of FoldX that includes new features like aromatic pi stacking and full pH dependence on protein stability, as well as improvements on different parameters. FoldX versions 4.1 and 5.1 predict both stabilizing and destabilizing mutations taking into consideration pH, temperature, ionic strength, waters, and metals. It contains different options that allow the user to perform alanine scanning, or multiple mutations at one position or at different positions. We believe this new version of FoldX will improve the applications of FoldX for various applications such as determining residue contribution to protein stability and binding, inferring disease-associated missense mutations, and engineering proteins with different stabilities and/or binding affinities.

## Supplementary Material

btaf064_Supplementary_Data
