# Supplementary material for: FoldX force field revisited, an improved version
Source: Bioinformatics. 2025 Feb 6;41(2):btaf064. doi: 10.1093/bioinformatics/btaf064 (PMC11879241; doi:10.1093/bioinformatics/btaf064)
Supplement: btaf064_Supplementary_Data [file btaf064_supplementary_data.zip › Supplementary.docx]

Supplementary Information

# Supplementary Methods

## ∆∆G Dataset calculations

### Structure Repair

Before modeling the mutants in the FSD, wild-type structures were repaired using RepairPDB FoldX’s command (using pH7, Ionic strength 0.05 and Temperature of 298 K). By this process FoldX identifies the residues that have unfavorable torsion angles, or VanderWaals clashes, or bad energy and repair the side chains in order to fix small crystallographic errors. RepairPDB was performed using the default parameters.

### Modeling of point mutations and ∆∆G calculation

After completing the repair process, each of the protein structures was mutated to all the single point mutations available in the FSD (Table S1), the command used was FoldX’s *BuildModel* command. This command mutates the residues to the indicated amino acid moving its neighboring residues and predicting changes in free energy upon mutation (∆∆G_mutation_ = ∆G_mutant_ - ∆G_wildtype_) in kcal/mol.

Throughout the calculations using versions v2 to v10, *BuildModel* was used consistently with the v1 repaired PDBs using default parameters. However, from v11R on, wildtype PDBs were side chain repaired using version v11.

The parameter *numberOfRuns* specifies the number of rotamer seed random generators when computing the mutants generating a slightly different structure per run. From v1 to v12 it was equal to 1 but we set it to 5 in order to calculate the mean (v11A) and the median (v11M) of the five structures generated with v11, allowing for a bigger rotamer space exploration while running *BuildModel* command.

## Helix Dipole

FoldX applies the Coulomb law to determine the electrostatic interaction dividing by the distance between charge (${charge}_{i}$) and dummy atom (${charge}_{e}$) (Ncap and Ccap for the dummy atoms at the N and C termini of the helix, respectively).

$$Constant=e^{-0.003414\left( Temperature-273 \right)}$$

$$Epss=\frac{332}{8.8\times Temperature\times Constant}$$

$$K=\sqrt{200\times abs\left( {charge}_{i} \times{charge}_{e} \right)\times\frac{0.05}{Temperature}}$$

$Electrostatic={charge}_{i}\times{charge}_{j}\times Epss\times\frac{e^{\left( -distance\times K \right)}}{{distance}^{2}}$ (eq. 1)

## Side chain entropy

In FoldX we used Abagyan´s entropic cost ($\Delta S_{Abagyan}$) of fixing amino acid side chains [(Abagyan and Totrov, 1994)](https://paperpile.com/c/IyK3rU/IepR).

$\Delta G=\Delta H+Temperature\times{\Delta S}_{Abagyan}$ (eq. 2)

## Pi-pi interactions

We determined the angle between the planes of the aromatics, His and/or guanidine rings ($angle$). We then assign an optimum interaction energy of 2.5 kcal/mol ($E_{opt}$) except for the interactions with Phe which are 0.25 weaker (in the case of two Phe, they are 0.5 kcal/mol weaker).

If one of the two residues of the interacting pair is His or Arg we only consider an angle (*angle*) between the two side chain planes (cyclic side chain of His and guanidinium group of Arg) when it is less than 30^o^ and then we apply equations 2 and 3 to determine the interaction energy ($E_{int}$).

$\vartheta=e^{-\frac{\left( \frac{angle}{100} \right)^{2}}{0.05}}$ $=e^{-\frac{{angle}^{2}}{500}}$ (eq. 3)

$E_{int}= \vartheta\times E_{opt}\times e^{-(\frac{({\delta_{\pi}-3.8)}^{2}}{0.2})}$ (eq. 4)

Otherwise, if the two residues involved are Phe, Tyr and/or Trp we use eq. 4 with $\vartheta$=1.

## pKa calculation

The environmental electrostatically modified pKa($pKa_{\varepsilon}$), is calculated using the following equation:

${pKa}_{\varepsilon}=pKa\left( \varepsilon\right)=pKa+\frac{\varepsilon}{Temperature\times2.3026}$ (eq. 5)

## Where ε is the sum of all the electrostatic interaction energies with all surrounding charged atoms. The pKa value has a negative sign for His, Lys, Arg and positive for Asp, Glu, Tyr and Cys.

## pH Dependency

Once we have determined the pKa of the charged groups of an aa we determined the percentage of ionization of the atom using the following equation:

$iondegree=\frac{1}{1+{10}^{({pKa}_{e}-pH)}}$ (eq. 6)

Electrostatic contribution of an atom to the free energy:

$E_{Electrostatic}=iondegree\times\varsigma\times\sum AtomElectrostatic$ (eq. 7)

Where $\varsigma is the the energy cost for ionization related to the solvent accessibility of the charged atom.$

## Buried volume calculation

To determine the percentage of the volume of an atom that is buried ($AccAtom$) we consider the atoms surrounding ($Satom$) our target atom in a sphere with radius smaller than 6Å, their volume ($Vol$) and the distance ($dist$) between the two atoms using eq. 8.

$AccAtom=\sum_{Satom} e^{-Vol\times\sqrt{\frac{dist}{24.5}}}$ (eq. 8)

Once we have calculated AccAtom, we determine the percentage of atom volume buried ($AtomBurial$) using eq. 9. We consider two experimental parameters per atom, Maximum atom volume burial ($AccMax$) and minimum atom volume burial ($AccMin$).

$AtomBurial=\frac{(AccAtom-AccMin)}{(AccMax-AccMin)}$ (eq. 9)

## AlphaFold2 modeling of mutants and wild types

To investigate whether the experimental ΔΔGs were predictable through the recent protein modeling tool AlphaFold2, we generated mutant and wild-type structures using the latest version of ColabFold (https://doi.org/10.1038/s41592-022-01488-1) with the following parameters: amber=True, num-recycle=15, num-models=5, model-type=auto, random-seed=16, use-gpu-relax=true. The sequences used by ColabFold were extracted from the original PDB structures used in the previous analysis. For each modeled protein variant, the top-ranking model from AlphaFold2 was chosen. We measured and extracted the FoldX stability (kcal/mol) and pLDDT score for all the models. For each mutant, we computed the variation of stability and pLDDT by subtracting the values from the respective wild-type models.

# Figures


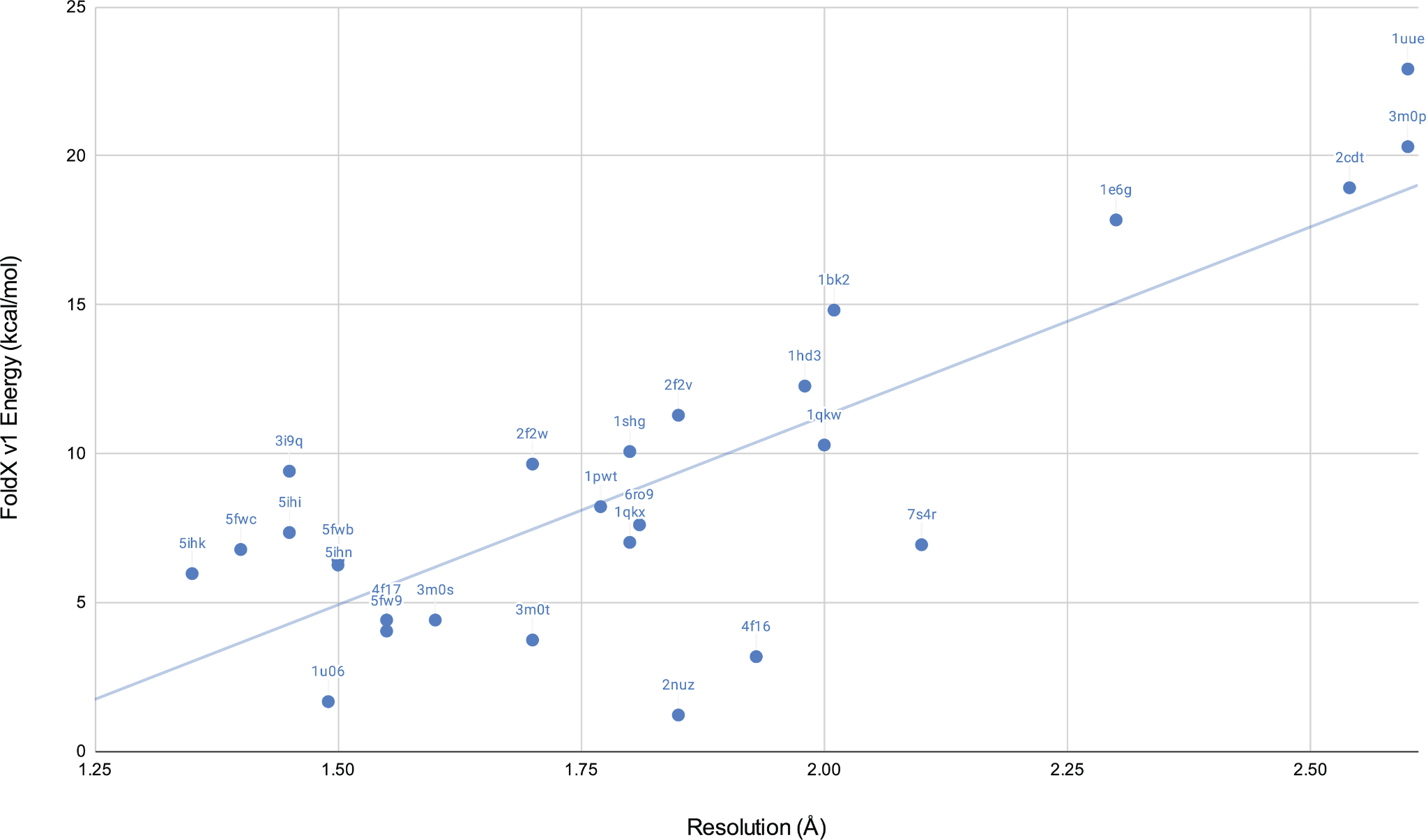


**Supp Figure 1: FoldXv1 Stability Energy in kcal/mol vs Resolution in Å, for 27 crystal structures of the SH3 domain.**


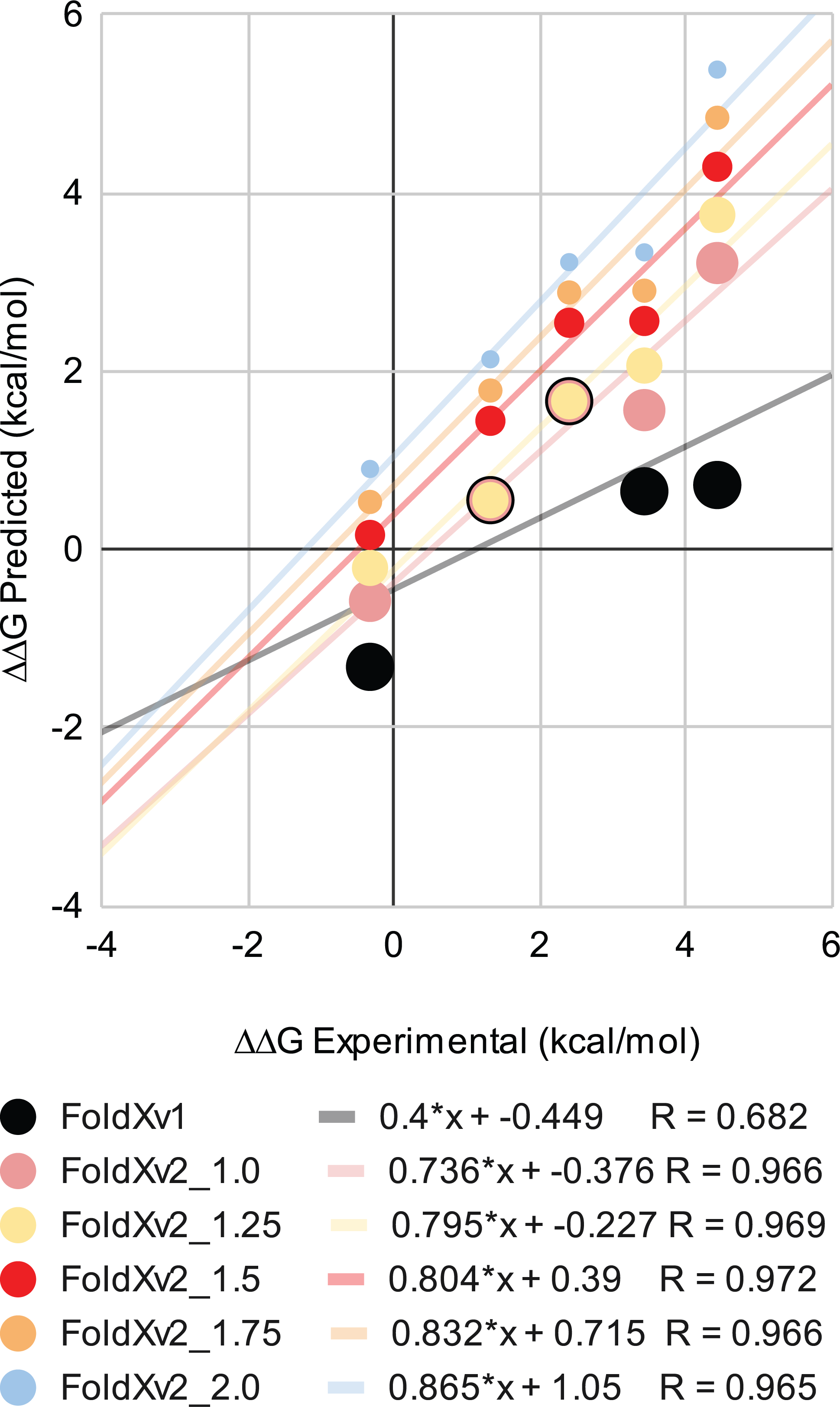


**Supp Figure 2: v1 vs v2 Pearson correlation and linear fit comparison between experimental and predicted ∆∆Gs for Calibration Subset of representative mutations for double salt bridges (Table S4). Equations obtained from the linear fitting are shown at the bottom of each plot with their corresponding R values.**


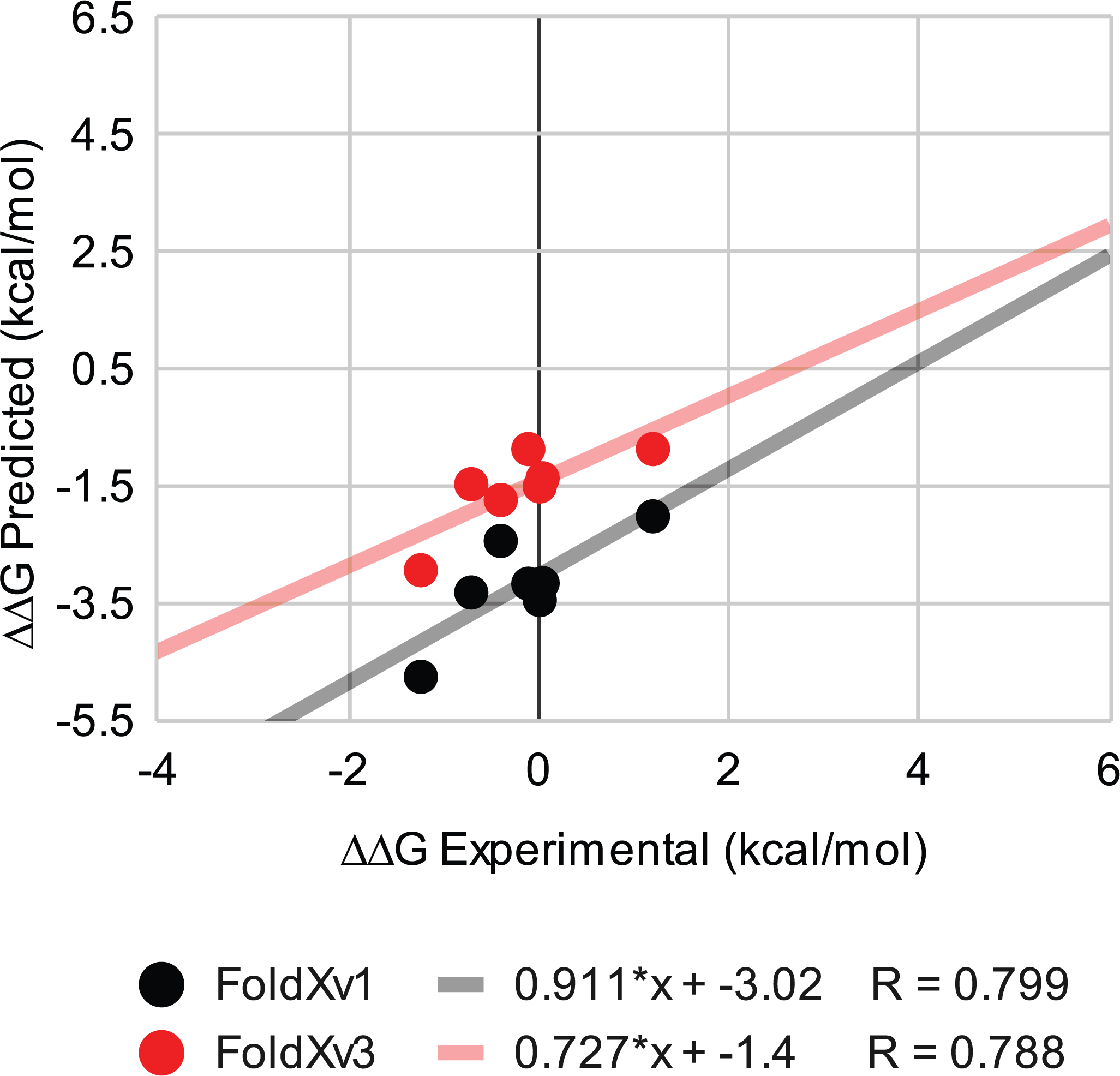


**Supp Figure 3: v2 vs v3 Pearson correlation and linear fit comparison between experimental and predicted ∆∆Gs for Calibration Subset of mutations that affect the Helix N-terminal dipole (Table S5. Equations obtained from the linear fitting are shown at the bottom of each plot with their corresponding R values.**

**
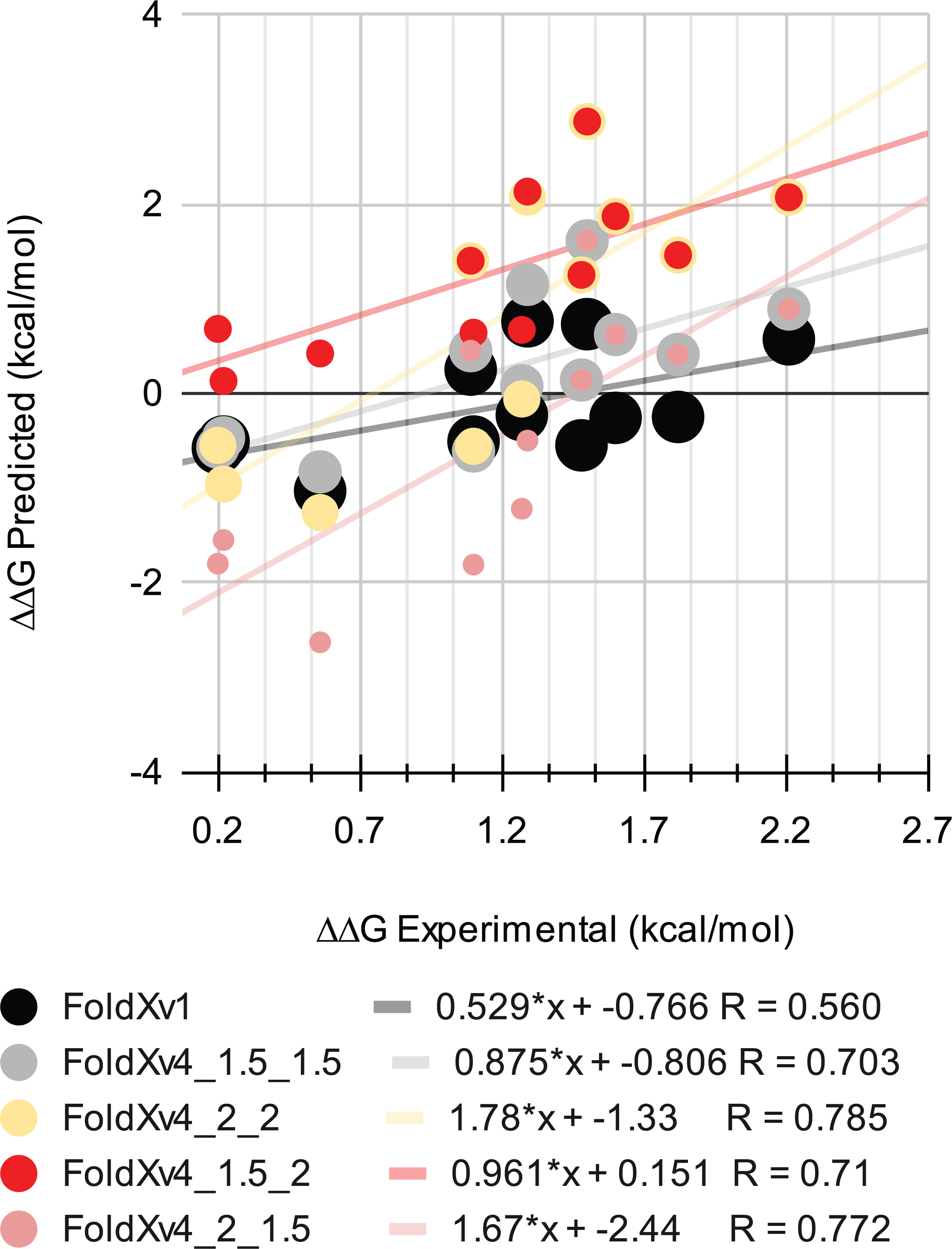
**

**Supp Figure 4: v1 vs v4 Pearson correlation and linear fit comparison between experimental and predicted ∆∆Gs for Calibration Subset of mutations representatives of N capping (Table S6). Equations obtained from the linear fitting are shown at the bottom of each plot with their corresponding R values.**


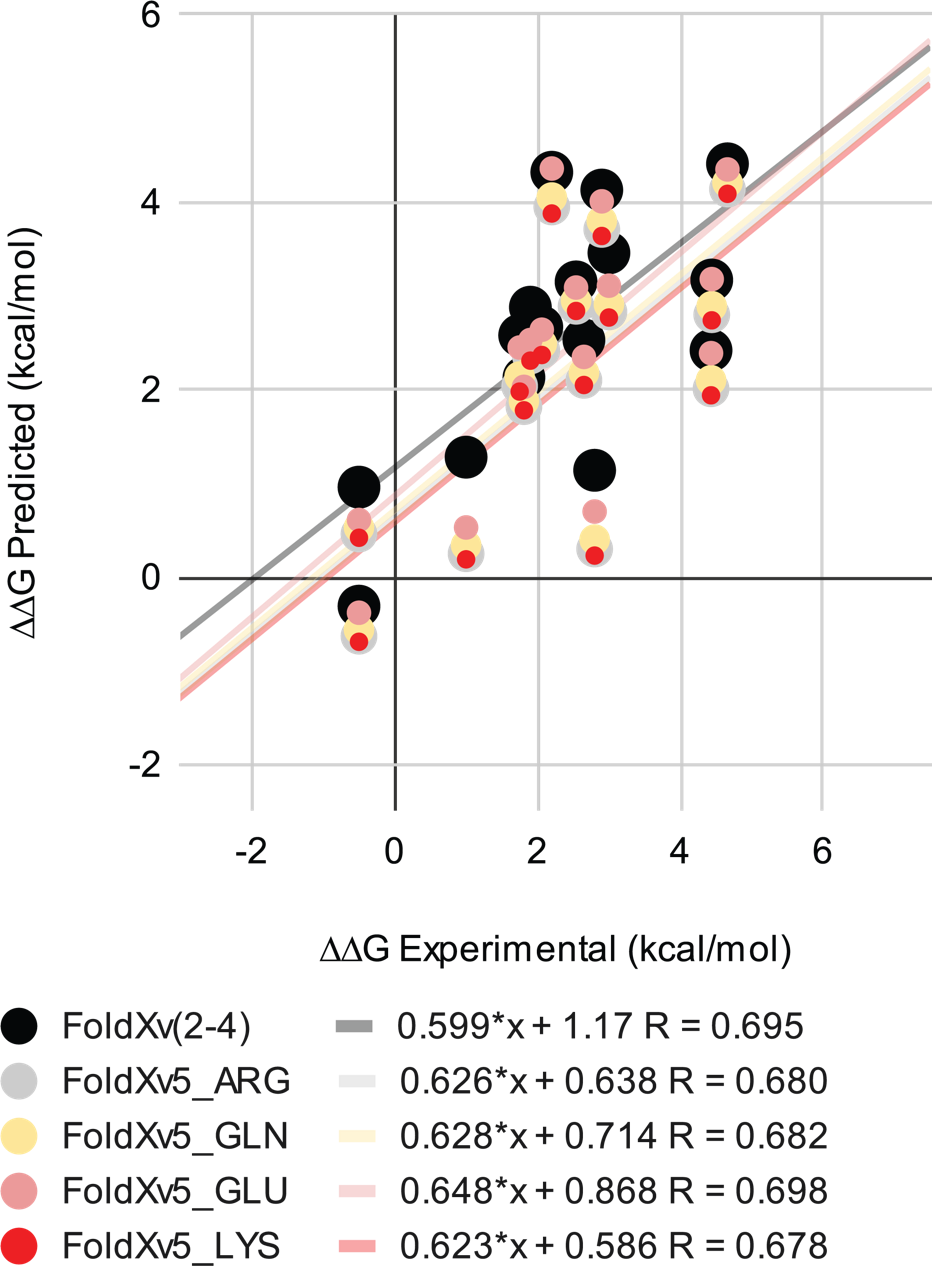


**Supp Figure 5: v1 vs v5 Pearson correlation and linear fit comparison between experimental and predicted ∆∆Gs for Calibration Subset of mutations representatives of Methionine having high side chain entropy (Table S7). Equations obtained from the linear fitting are shown at the bottom of each plot with their corresponding R values.**

**
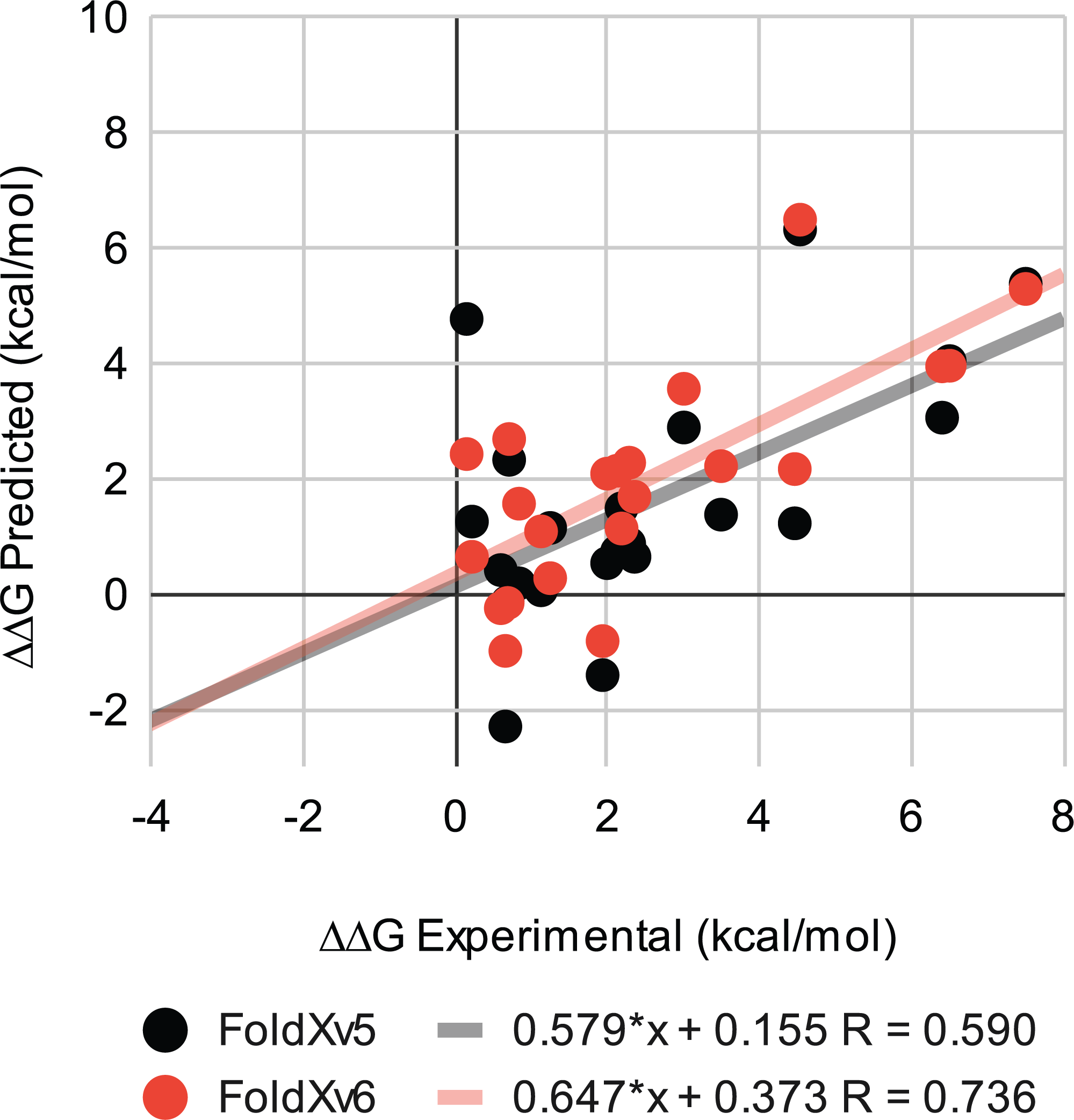
**

**Supp Figure 6: v5 vs v6 Pearson correlation and linear fit comparison between experimental and predicted ∆∆Gs for Calibration Subset of mutations representatives of PI-PI interaction (S8 Table). Equations obtained from the linear fitting are shown at the bottom of each plot with their corresponding R values.**


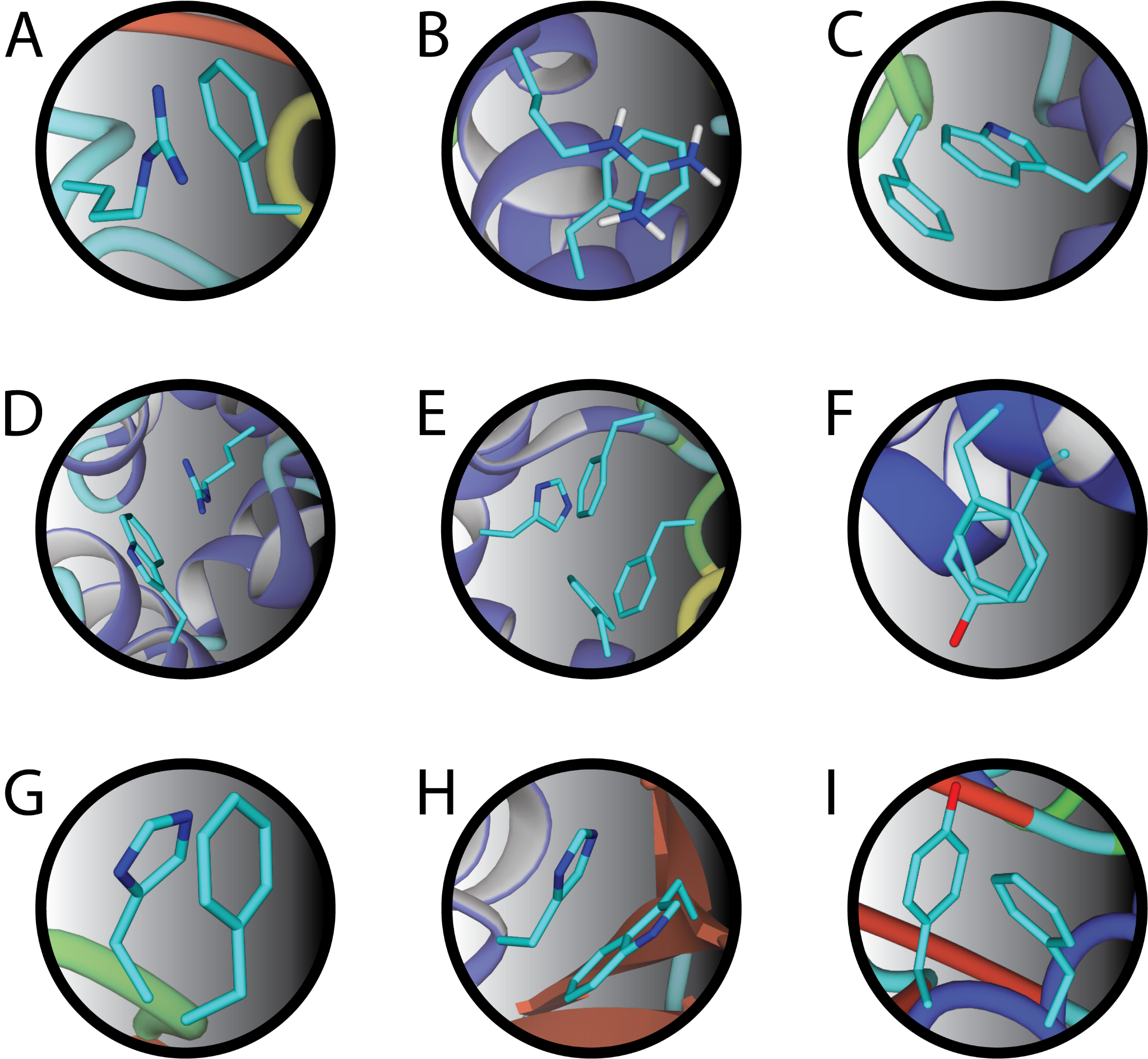


**Supp Figure 7: Examples of PI-PI Interactions: (A,B) Phenylalanine-Arginine pi-pi interaction; (C) Tryptophan-Phenylalanine; (D) Tryptophan-Arginine; (E) Aromatic cluster; (F) Phenylalanine-Tyrosine; (G) Histidine-Phenylalanine; (H) Histidine-Tryptophan; (I) perpendicular Phenylalanine-Tyrosine.**

**
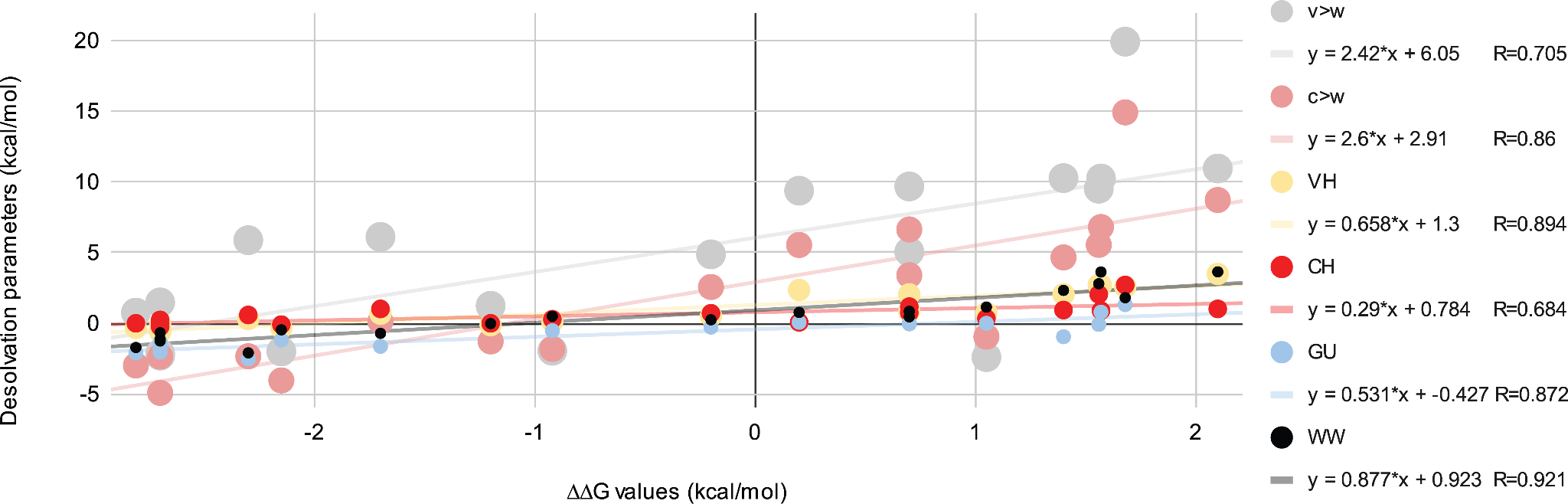
**

**Supp Figure 8: FoldX correlation analysis between desolvation parameters for the 19 aa versus the theoretical and estimated values shown (Table S10). v>w: Side-chain Kd values for side-chain transfer from water to vapor** [(Wolfenden *et al.*, 1981)](https://paperpile.com/c/JnL2lx/y5JM)**. c>w: Side-chain Kd values for transfer from water to cyclohexane** [(Radzicka *et al.*, 1988)](https://paperpile.com/c/JnL2lx/Tosg)**. VH: Tendency of amino acid residue to be found in a transmembrane helix** [(Hessa *et al.*, 2005)](https://paperpile.com/c/JnL2lx/cVft)**. CH: Tendency of amino acid residue to be buried in the interior of a globular protein** [(Chothia, 1976)](https://paperpile.com/c/JnL2lx/5tq3) **GU: Amino acid side-chain Kd values for transfer from water to wet octanol** [(Guy, 1985)](https://paperpile.com/c/JnL2lx/kmE8)**. WW: Theoretical pentapeptide Kd values for transfer from water to wet octanol, after adjustment for the estimated effects of occlusion by neighboring residues** [(Wimley *et al.*, 1996)](https://paperpile.com/c/JnL2lx/tqgR)**.**

**
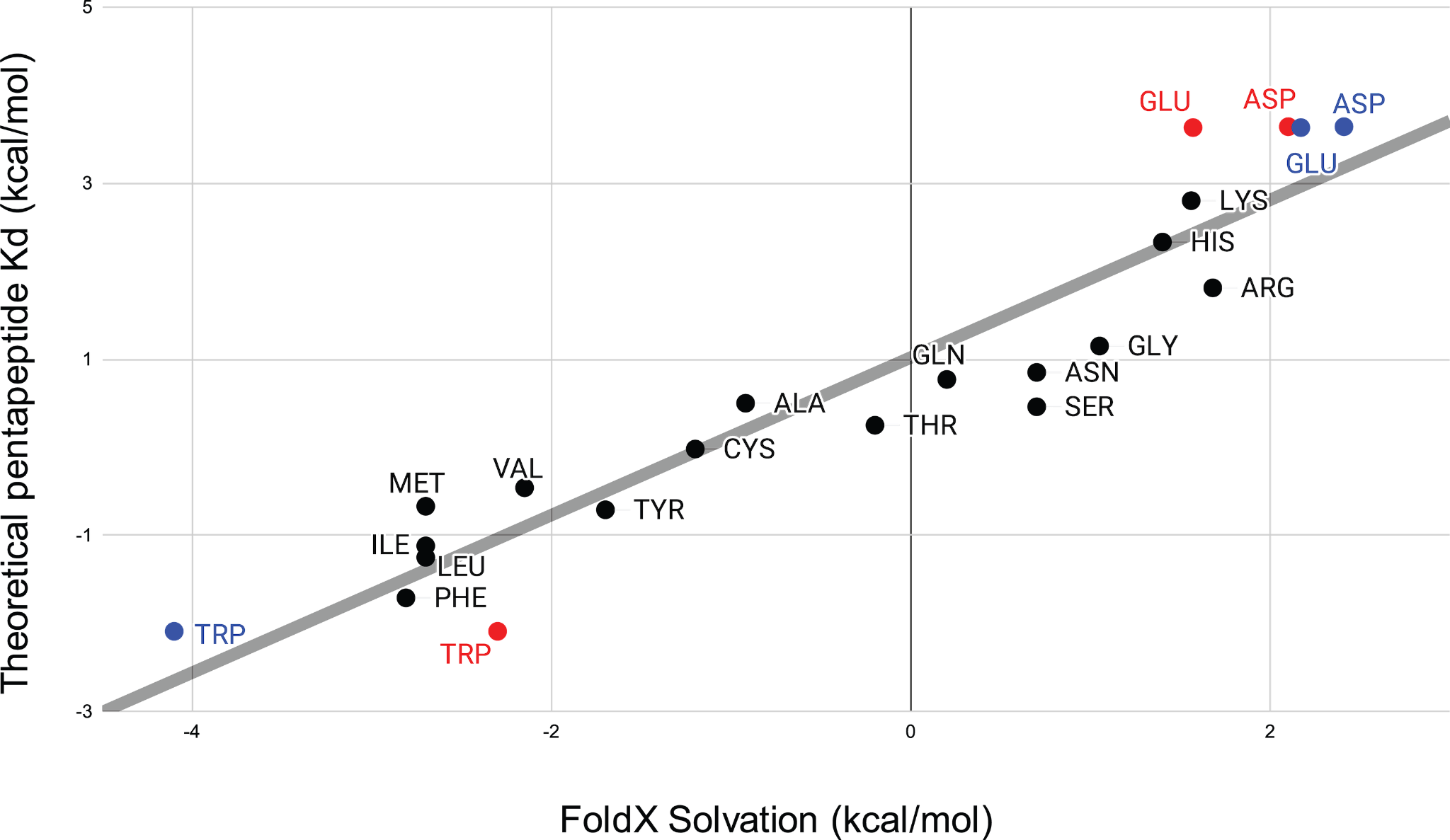
**

**Supp Figure 9: FoldX Correlation analysis between desolvation parameters for the 19 aa versus the theoretical pentapeptide Kd values for transfer from water to wet octanol, after adjustment for the estimated effects of occlusion by neighboring residues** [(Wimley *et al.*, 1996)](https://paperpile.com/c/JnL2lx/tqgR) **(Table S10). In red (Asp, Glu, Trp), Kd values for transfer from water to wet octanol deviating from the ideal correlation, in blue (Asp, Glu, Trp), Kd values for transfer from water to wet octanol after hydrophobic desolvation parameter modification.**

**
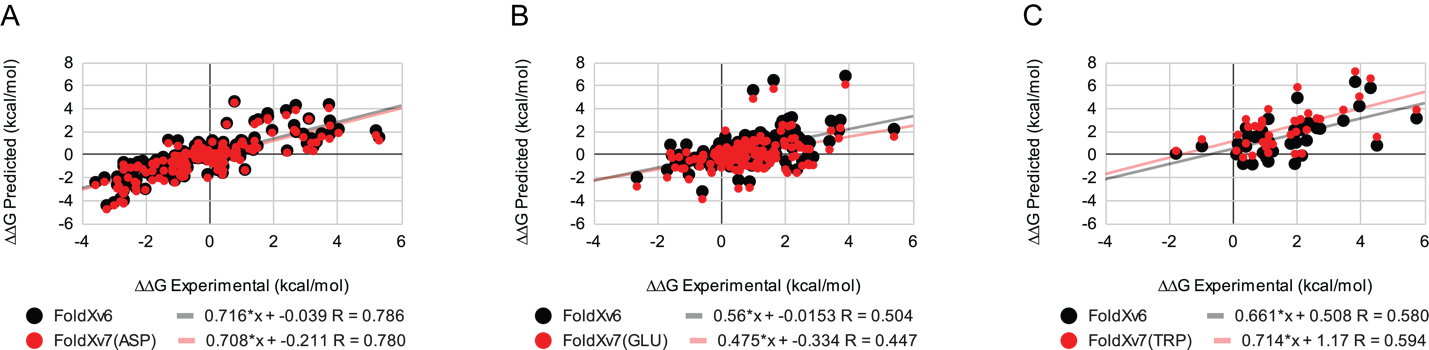
**

**Supp Figure 10: v6 vs v7 Pearson correlation and linear fit comparison between experimental and predicted ∆∆Gs A) DX subset of asp mutations to any amino acid; B) EX subset of glu mutations to any amino acid; C) WX subset of trp mutations to any amino acid; Equations obtained from the linear fitting are shown at the bottom of each plot with their corresponding R values.**

**
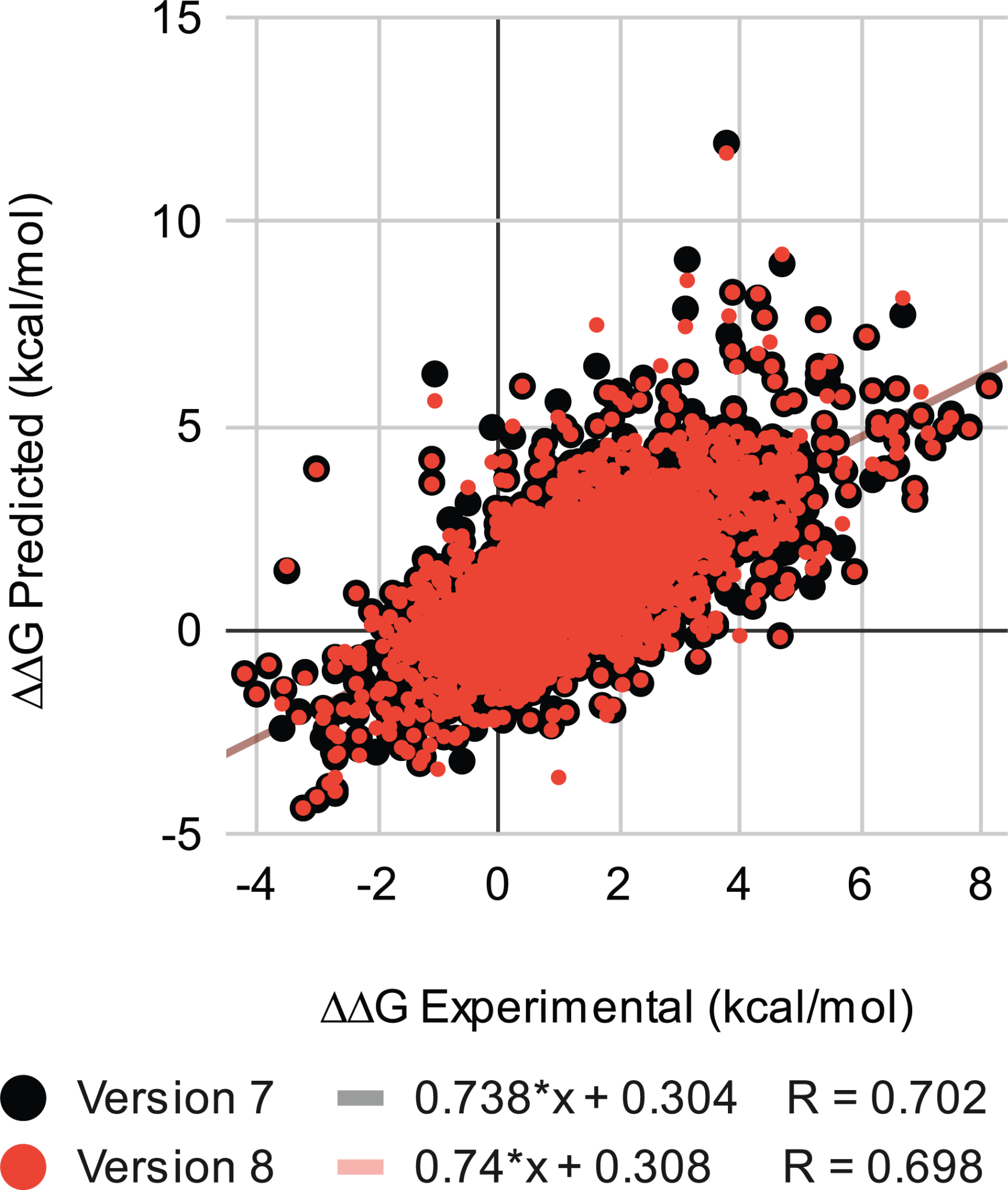
**

**Supp Figure 11: Correlation comparison between v7 and v8 between experimental and predicted ∆∆G for 2484 mutations. Equations obtained from the linear fitting are shown at the bottom of the figure with their corresponding R values.**

**
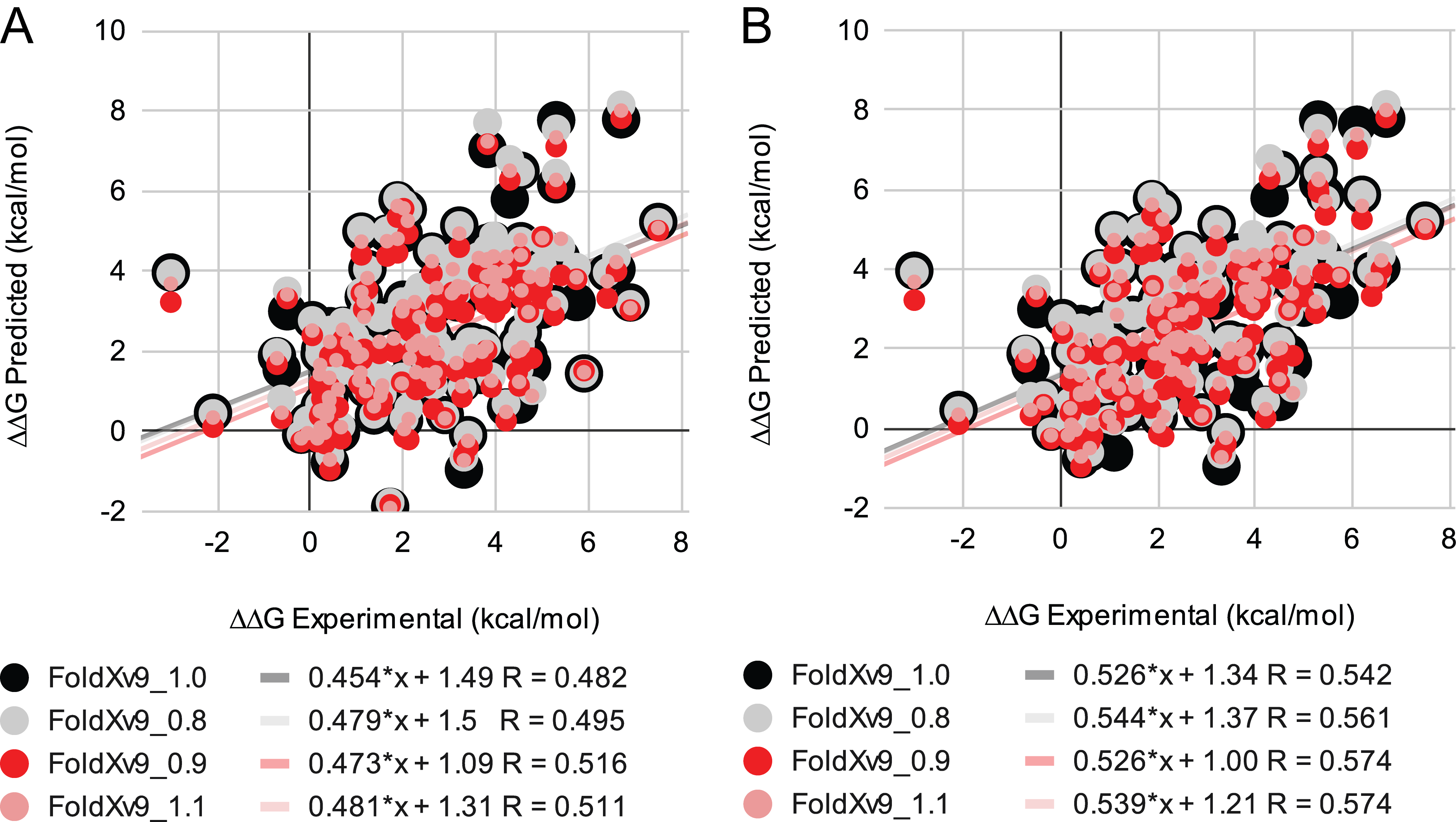
**

**Supp Figure 12: Correlation comparison between v8 and v9 between experimental and predicted ∆∆Gs. A) 130 mutation (Table S14) random training subset for the different aromatic volume multiplying factors (0.8, 0.9, 1, 1.1); B ) 131 mutation (Table S14) random validation subset for the different aromatic volume multiplying factors (0.8, 0.9, 1, 1.1); Equations obtained from the linear fitting are shown at the bottom of each plot with their corresponding R values.**

**
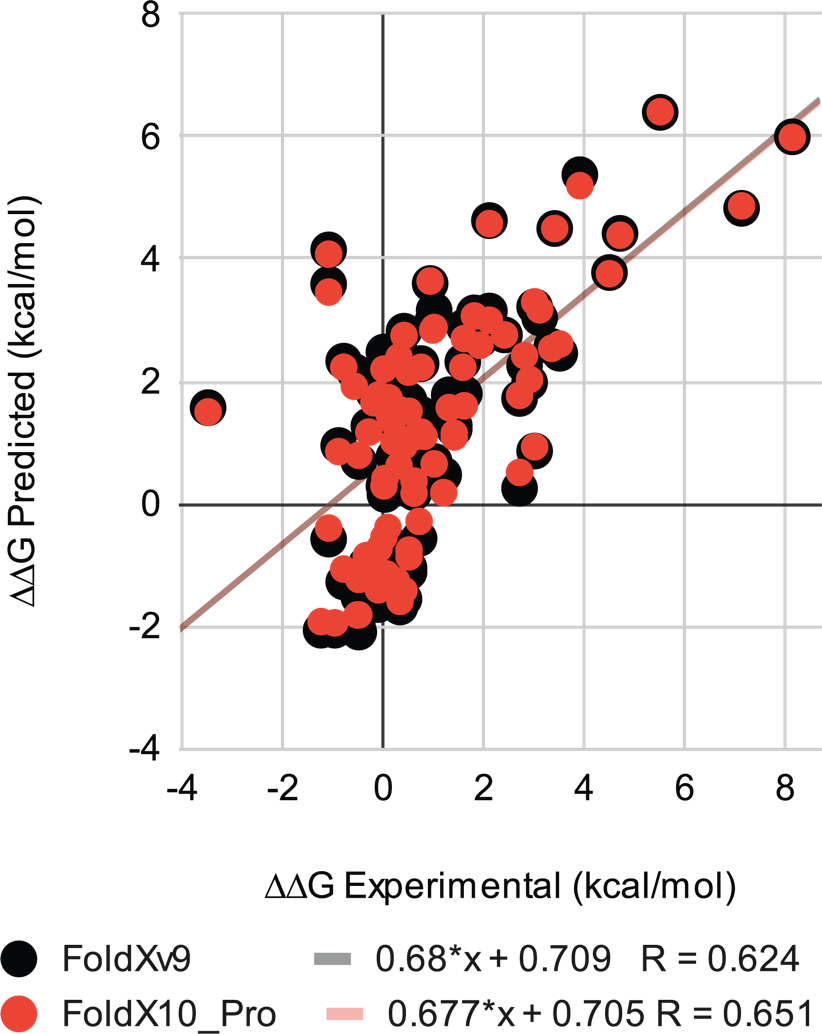
**

**Supp Figure 13: Correlation comparison between v9 and v10_Pro between experimental and predicted ∆∆Gs for Mutations involving Pro. Equations obtained from the linear fitting are shown at the bottom of the plot with its corresponding R values.**

**
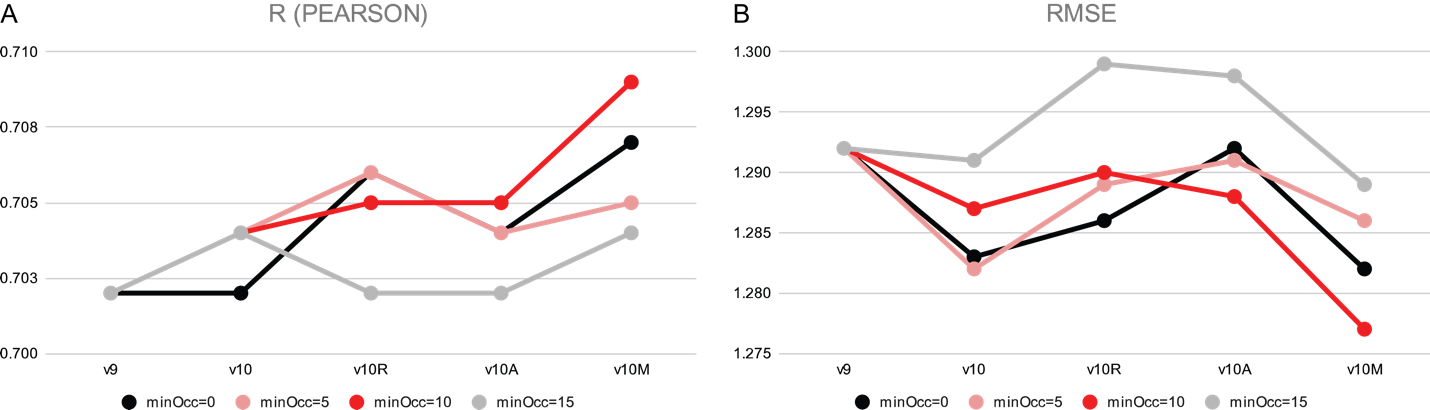
**

**Supp Figure 14: A) Pearson correlation values for v9, v10, v10R, v10A, and v10M using different minimum atomic occupancies corrections (0, 5, 10, or 15); B) RMSE values for v9, v10, v10R, v10A, and v10M using different minimum atomic occupancies corrections (0, 5, 10, or 15).**

References

[Chothia,C. (1976) The nature of the accessible and buried surfaces in proteins. *J. Mol. Biol.*, **105**, 1–12.](http://paperpile.com/b/JnL2lx/5tq3)

[Guy,H.R. (1985) Amino acid side-chain partition energies and distribution of residues in soluble proteins. *Biophys. J.*, **47**, 61–70.](http://paperpile.com/b/JnL2lx/kmE8)

[Hessa,T. *et al.* (2005) Recognition of transmembrane helices by the endoplasmic reticulum translocon. *Nature*, **433**, 377–381.](http://paperpile.com/b/JnL2lx/cVft)

[Radzicka,A. *et al.* (1988) Influences of solvent water on protein folding: free energies of solvation of cis and trans peptides are nearly identical. *Biochemistry*, **27**, 4538–4541.](http://paperpile.com/b/JnL2lx/Tosg)

[Wimley,W.C. *et al.* (1996) Solvation energies of amino acid side chains and backbone in a family of host-guest pentapeptides. *Biochemistry*, **35**, 5109–5124.](http://paperpile.com/b/JnL2lx/tqgR)

[Wolfenden,R. *et al.* (1981) Affinities of amino acid side chains for solvent water. *Biochemistry*, **20**, 849–855.](http://paperpile.com/b/JnL2lx/y5JM)
